# Insight into the molecular requirements for pathogenicity of *Fusarium oxysporum *f. sp. *lycopersici *through large-scale insertional mutagenesis

**DOI:** 10.1186/gb-2009-10-1-r4

**Published:** 2009-01-09

**Authors:** Caroline B Michielse, Ringo van Wijk, Linda Reijnen, Ben JC Cornelissen, Martijn Rep

**Affiliations:** 1 Plant Pathology, Swammerdam Institute for Life Sciences, University of Amsterdam, Kruislaan 318, 1098 SM Amsterdam, The Netherlands; 2 Current address: Plant Physiology, Swammerdam Institute for Life Sciences, University of Amsterdam, Kruislaan 318, 1098 SM Amsterdam, The Netherlands

## Abstract

**Background:**

*Fusarium oxysporum *f. sp. *lycopersici *is the causal agent of vascular wilt disease in tomato. In order to gain more insight into the molecular processes in *F. oxysporum *necessary for pathogenesis and to uncover the genes involved, we used *Agrobacterium*-mediated insertional mutagenesis to generate 10,290 transformants and screened the transformants for loss or reduction of pathogenicity.

**Results:**

This led to the identification of 106 pathogenicity mutants. Southern analysis revealed that the average T-DNA insertion is 1.4 and that 66% of the mutants carry a single T-DNA. Using TAIL-PCR, chromosomal T-DNA flanking regions were isolated and 111 potential pathogenicity genes were identified.

**Conclusions:**

Functional categorization of the potential pathogenicity genes indicates that certain cellular processes, such as amino acid and lipid metabolism, cell wall remodeling, protein translocation and protein degradation, seem to be important for full pathogenicity of *F. oxysporum*. Several known pathogenicity genes were identified, such as those encoding chitin synthase V, developmental regulator FlbA and phosphomannose isomerase. In addition, complementation and gene knock-out experiments confirmed that a glycosylphosphatidylinositol-anchored protein, thought to be involved in cell wall integrity, a transcriptional regulator, a protein with unknown function and peroxisome biogenesis are required for full pathogenicity of *F. oxysporum*.

## Abstract 2

An insertional mutagenesis screen identifies pathogenicity-related genes in the plant fungal pathogen *Fusarium oxysporum*.

## Background

*Fusarium oxysporum*, a soil-borne facultative pathogen with a worldwide distribution, causes vascular wilt and foot-, root-, and bulbrot diseases in a wide variety of economically important crops [1,2]. *F. oxysporum *isolates are highly host-specific and have been grouped into *formae speciales *according to their host range [1]. Recently, *F. oxysporum *has also been reported as an emerging human pathogen, causing opportunistic mycoses [3-5].

Over the years numerous studies have been performed to understand *F. oxysporum*-mediated disease development. The process of vascular infection has been studied using light, fluorescence and electron microscopy and can be divided into several steps: root recognition, root surface attachment and colonization, penetration of the root cortex, and hyphal proliferation within the xylem vessels. This hyphal proliferation in vessels causes characteristic disease symptoms, such as vein clearing, leaf epinasty, wilt and defolation, eventually leading to death of the host plant. At this stage, *F. oxysporum *invades the parenchymatous tissue and starts sporulating on the plant surface, thereby completing its pathogenic life cycle [6].

Forward and reverse genetics have improved our understanding of molecular mechanisms involved in pathogenesis. Targeted deletion of genes encoding a mitogen-activated protein kinase (*fmk1*) and G-protein subunits α (*fga1*, *fga2*) and β (*fgb1*) revealed that mitogen-activated protein kinase (MAPK) and cyclic AMP-protein kinase A (cAMP-PKA) cascades both regulate virulence in *F. oxysporum *[7-11]. In addition, several genes necessary for maintenance of cell wall integrity and full virulence have been identified - encoding chitin synthases (*chs2*, *chs7*, *chsV*, and *chsVb*), a GTPase (*rho1*), and a β-1,3-glucanosyltransferase (*gas1*) - and it has been postulated that cell wall integrity might be necessary for invasive growth and/or resistance to plant defense compounds [12-16]. The degree to which cell wall degrading enzymes contribute to the infection process is not yet fully understood. It has been described that *Fusarium *secretes an array of cell wall degrading enzymes, such as polygalacturonases, pectate lyases, xylanases and proteases, during root penetration and colonization [2]. However, inactivation of individual cell wall degrading enzyme- or protease-encoding genes (for example, pectate lyase gene *pl1*, xylanase genes *xyl3*, *xyl4*, and *xyl5*, polygalacturonase genes *pg1*, *pg5*, and *pgx4*, and the subtilase gene *prt1 *[6,17-23]) did not have a detectable effect on virulence. Deletion of *xlnR*, which encodes the transcriptional activator XlnR, a regulator of the expression of many xylanolytic and cellulolytic genes, had no effect on virulence either, although expression of xylanase genes was strongly reduced [24]. On the other hand, targeted disruption of the carbon catabolite repressor SNF1 did result in reduced expression of several cell wall degrading enzymes and virulence [25], indicating that carbon catabolite repression and, thus, adaptation of the central carbon metabolism plays a role in pathogenicity.

Also, nitrogen regulation was shown to be important for the infection process. Inactivation of the global nitrogen regulator Fnr1 abolished the expression of nutrition genes normally induced during the early phase of infection, and resulted in reduced pathogenicity [26]. Finally, various genes with diverse functions have been identified to play a role in pathogenicity, including those encoding a pH responsive transcription factor (*pacC*), a Zn(II)2Cys6 transcriptional regulator (*FOW2*), argininosuccinate lyase (*ARG1*), a mitochondrial carrier protein (*FOW1*), an F-box protein (*FRP1*), a secreted protein (*SIX1*), a chloride channel (*CLC1*), and a chloride conductance regulatory protein (*FPD1*) [27-33].

The majority of the above-mentioned genes have been identified and studied based on the function of homologous genes in other organisms. To uncover genes necessary during pathogenesis in an unbiased manner, insertional mutagenesis has been used for a number of fungal plant pathogens [34,35]. This approach has also been applied to *F. oxysporum*, although only a limited number of insertion mutants were generated using restriction enzyme mediated insertion (REMI) or random plasmid DNA insertion and only a small number of pathogenicity genes have been identified in this way [13,28-30,32].

In order to identify many more genes important for the ability of *F. oxysporum *to cause disease, and thus to gain a more global understanding of the infection process, we used an *Agrobacterium*-mediated insertional mutagenesis approach. This approach has been successfully used with other plant pathogenic fungi, like *Magnaporthe oryzae*, *M. grisea *and *Leptosphaeria maculans*, to generate large insertional mutant collections and to identify pathogenicity genes [36-38]. In this study, a collection of more than 10,000 transformants of *F. oxysporum *f. sp. *lycopersici *was generated, and each transformant was tested for loss of pathogenicity. To estimate the probability that a transfer DNA (T-DNA) insertion is linked to the pathogenicity phenotype and since downstream analysis is facilitated by single T-DNA integrations, Southern analysis and thermal asymmetric interlaced PCR (TAIL-PCR) were performed on all pathogenicity mutants. The outcome was used to determine T-DNA copy number and integration patterns and to identify potential pathogenicity genes. Predicted functions of potential pathogenicity genes allowed tentative identification of molecular processes required for pathogenesis. For five genes predicted to be involved in some of these processes, involvement in pathogenicity was verified by complementation and gene knock-out studies.

## Results

### Identification of pathogenicity mutants from a collection of *F. oxysporum *transformants

**Figure 1:**
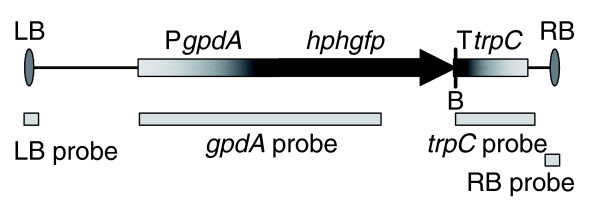
**T-DNA of pPK2 *hphgfp***. LB, left border; P*gpdA*, *Aspergillus nidulans *glyceraldehyde-3-phosphate dehydrogenase promoter; *hphgfp*, translational fusion of hygromycin B resistance gene and green fluorescent protein gene; T*trpC*, *A. nidulans trpC *terminator; RB, right border; B, *Bam*HI restriction site.

A collection of 10,290 transformants was generated through *Agrobacterium*-mediated transformation using the T-DNA of pPK2*hphgfp *as insertional mutagen (Figure 1). All transformants were assessed for loss of pathogenicity in root-dip bioassays using three seedlings per transformant. Transformants displaying an apparent loss or strong reduction of pathogenicity were re-tested, again using three seedlings per transformant. In this way, out of the 10,290 transformants, 145 putative pathogenicity mutants were identified. Subsequently, these mutants were assessed in a third root-dip bioassay using 20 plants per mutant followed by statistical analysis. This resulted in the identification of 106 mutants with a reproducible pathogenicity defect. The pathogenicity mutants were classified according to severity of pathogenicity loss, based on the average disease index. The average disease index caused by the wild-type parent strain (4287) was 3 ± 0.6 (based on 13 independent bioassays). In total, 20 mutants were classified as non-pathogenic (class 1, disease index = 0), 47 mutants were severely reduced in pathogenicity (class 2, disease index <1) and 39 mutants were reduced in pathogenicity (class 3, disease index ≥ 1, but still statistically different compared to the wild-type infection at a 5% confidence interval) (Table 1). Thus, 1% of the entire collection of transformants was (severely) reduced in pathogenicity or totally non-pathogenic on tomato seedlings.

**Table 1:** Classification of pathogenicity phenotypes

Class	Pathogenicity phenotype	Number of pathogenicity mutants	Number of growth mutants
1	Non-pathogenic (DI = 0)	20	12
2	Severely reduced (DI <1)	47	25
3	Reduced (DI ≥ 1)	39	9
	Total	106	46

DI, disease index.

### Phenotypic characterization of the pathogenicity mutants

The growth phenotype on various carbon sources of each pathogenicity mutant was determined to assess whether the reduced pathogenicity phenotype could be attributed to a metabolic defect. All mutants were grown for seven days on potato dextrose agar (PDA), Czapek-Dox agar (CDA) and minimal medium containing as sole carbon source either sucrose, glycerol, ethanol, malic acid or citric acid. Aberrant growth phenotypes among the mutants varied from slightly to severely reduced growth on one or several of the media tested, slightly reduced growth on all media tested, severely reduced growth on all media tested to no growth on any media tested except PDA. In total, 60 of the 106 pathogenicity mutants displayed no aberrant growth phenotype. The frequency and severity of growth phenotypes of the remaining 46 mutants tended to increase with increased reduction of pathogenicity: from the mutant classes 1, 2, and 3, respectively, 60%, 53%, and 23% of the mutants showed a growth phenotype different from the wild-type strain (Table 1). Nevertheless, eight mutants with no detectable growth phenotype still showed complete loss of pathogenicity (class 1).

### Analysis of T-DNA integration patterns

**Figure 2:**
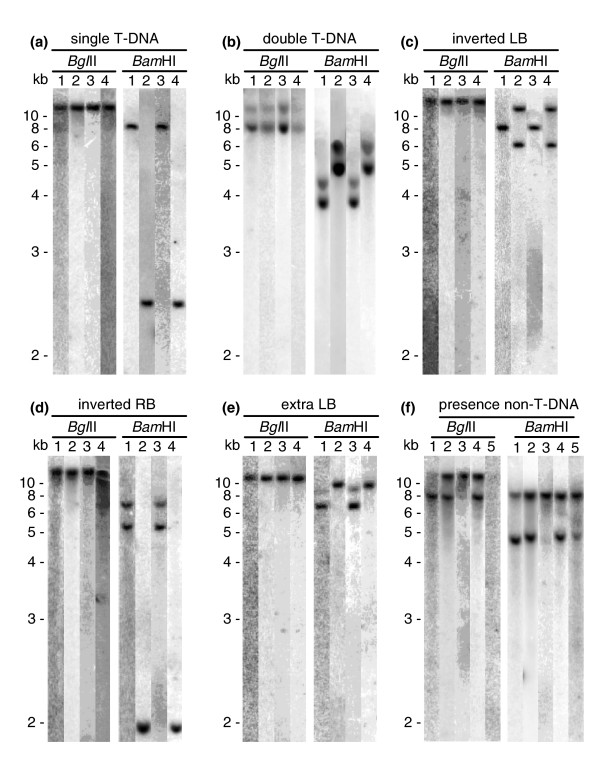
**T-DNA integration patterns in pathogenicity mutants**. **(a) **Representative transformant with a single T-DNA integration, resulting in one fragment with either restriction enzyme or probe (lanes 1 and 2). **(b) **Representative transformant with a double unlinked T-DNA integration, resulting in two fragments with either restriction enzyme or probe (lanes 1 and 2). **(c) **Representative transformant with a double inverted T-DNA integration fused at the LB, resulting in one fragment in the *Bgl*II digestion hybridized with the *gpdA *or *trpC *probe (*Bgl*II, lanes 1 and 2), one fragment of 8.4 kb in the *Bam*HI digestion when hybridized with the *gpdA *probe (*Bam*HI, lane 1) and two fragments when hybridized with the *trpC *probe (*Bam*HI, lane 2). **(d) **Representative transformant with a double inverted T-DNA integration fused at the RB, resulting in one fragment in the *Bgl*II digestion when hybridized with the *gpdA *or *trpC *probe (*Bgl*II, lanes 1 and 2), a fragment of 2.2 kb in the *Bam*HI digestion when hybridized with the *trpC *probe (*Bam*HI, lane 2) and two fragments when hybridized with the *gpdA *probe (*Bam*HI, lane 1). **(e) **Representative transformant with a single T-DNA integration and a second aborted T-DNA integration event. **(f) **Representative transformant with more than one T-DNA and with binary vector DNA. Blots were hybridized with *gpdA *(lane 1), *trpC *(lane 2), LB (lane 3), RB (lane 4) and pPZP (lane 5) probes. This figure is a composition of different blots, which results in minor differences in apparent fragment sizes. The negative results for the pPZP probe for the transformants depicted in (b-f) are omitted for clarity.

To assess the general characteristics of T-DNA integration into the genome of *F. oxysporum *and to facilitate selection of mutants for further analysis, all mutants were analyzed for T-DNA copy number, mode of T-DNA integration (tandem or inverted repeats), abortive T-DNA integration events, and the presence of non-T-DNA (binary vector). In order to discriminate between these various modes of T-DNA integration the chromosomal DNA was cut with *Bgl*II, which does not cut within the T-DNA, or *Bam*HI, which cuts once in the T-DNA, and hybridized with five different probes, notably the gpdA, trpC, left border (LB), right border (RB) and binary vector probes (Figure 1a). Examples of various T-DNA integration patterns observed are depicted in Figure 2a-f. In case of a single T-DNA integration, one fragment is observed with either restriction enzyme or probe (Figure 2a, lanes 1 and 2). A double T-DNA integration could result in two integration events at two different chromosomal locations, resulting in two fragments with either restriction enzyme (Figure 2b, lanes 1 and 2). A double T-DNA integration can also occur at one chromosomal location; in this case the T-DNA could be integrated as a tandem repeat, fused at the LB (Figure 2c), or at the RB (Figure 2d). An abortive T-DNA integration event or the presence of non-T-DNA will result in additional fragments in hybridizations using specific border or binary vector probes, respectively (Figure 2e,g lanes 3-5).

Taken together, Southern analysis performed on 99 of the 106 pathogenicity mutants revealed that 65, 28, and 6 of the mutants contained single, double or multiple (three or more) T-DNA insertions, respectively. This translates to an average T-DNA copy number of 1.4. Non-T-DNA was present in 4% of the pathogenicity mutants. These numbers are comparable to those of the entire transformant collection (a random subset of 72 out of the 10,290 transformants was analyzed in the same way; data not shown). In 22 of the mutants in which a double T-DNA integration had occurred, the two T-DNA copies had integrated in close proximity of each other or exactly at the same chromosomal location, leading to inverted repeats. Only in six mutants with two insertions had the T-DNAs integrated in two different chromosomal locations. Finally, based on the results obtained with the LB and RB probes, we concluded that a LB or RB truncation had occurred in 13% and 1% of the mutants analyzed, respectively. An additional LB or RB was observed in 5% of the mutants. There was no correlation between severity of pathogenicity loss and the number of T-DNA insertions (data not shown).

In conclusion, the majority of the mutants carried either a single T-DNA or a double T-DNA integrated at a single chromosomal position. In addition, no major differences with regards to the T-DNA copy number and integration pattern were observed between the pathogenicity mutants and a random subset of the entire transformant collection.

### Isolation of T-DNA flanking regions

TAIL-PCR was carried out on all pathogenicity mutants to isolate DNA sequences flanking the T-DNA. Using several different degenerated primers, LB and RB flanking sequences were obtained for, respectively, 74% and 84% of the pathogenicity mutants. In total, 89 LB and 109 RB flanking sequences were isolated. From these LB and RB flanking sequences, 20% and 18%, respectively, corresponded to binary vector backbone or other T-DNA sequences. Another 1% of the LB and 4% of the RB flanking sequences were identical to multiple genomic regions, suggesting that the corresponding T-DNAs were integrated into repetitive sequences. Excluding these, in total, 70 unique LB and 85 unique RB flanking sequences were obtained. By comparison to the genome sequence of *F. oxysporum *[39], these unique sequences allowed the localization of the T-DNA integration sites and identification of putative genes affected by these integration events.

### Deletions and rearrangements

Comparison of Southern and TAIL-PCR data enabled us to identify complex T-DNA integration events. For 22% of the pathogenicity mutants, genomic sequences flanking both the LB and RB were isolated. This revealed that, in most cases, several nucleotides (4-131 bp) were deleted at the insertion site. For four additional mutants, the LB and RB sequences were not located in close proximity of each other in the genome sequence, even though Southern analysis clearly indicated that these mutants carry a single T-DNA. In all four cases the isolated T-DNA flanking regions were located on the same supercontig, but several kilobases apart from each other. For two of these mutants we could confirm by PCR that a region of 4,210 or 9,826 bp was deleted at the insertion site (data not shown), leading to deletion of a complete open reading frame (ORF; *FOXG_08594 *and *FOXG_10510*, respectively). Chromosomal translocations or inversions were deduced for five pathogenicity mutants. For two mutants such a chromosomal rearrangement (translocation) could be confirmed by PCR. These rearrangements were specific for the mutants, as they were absent in the parental strain (data not shown). Finally, for four of the pathogenicity mutants more T-DNA borders were identified in the TAIL-PCR than was expected based on the Southern analysis. These borders were integrated in close proximity of each other (<2.5 kb) and their presence could, therefore, have been missed in the Southern analysis due to the choice of restriction enzymes.

### Identification of putative pathogenicity genes

Only when a T-DNA had integrated in an ORF, or within 1,000 bp up- or downstream of an ORF, was it assumed that the expression of that gene could be influenced by the T-DNA insertion and the gene was designated as potentially involved in pathogenicity. The T-DNA insertions were grouped based on the distance to the nearest ORF: within an ORF;within 500 bp upstream or 200 bp downstream of an ORF; within 1,000-500 bp upstream or 200-1,000 bp downstream of an ORF; and within 'intergenic regions' (3,000-1,000 bp up- or downstream of an ORF). The majority of the insertions (62) were found in an ORF (Additional data file 1). Of the remaining insertions, 25 (Additional data file 2) were integrated within 500 bp upstream or 200 bp downstream of an ORF and 27 (Additional data file 3) within 1,000-500 bp upstream or 200-1,000 bp downstream of an ORF. A minority of the T-DNA insertions (11) was located in intergenic regions (Additional data file 4). Finally, a remaining set of seven T-DNA insertions was integrated at a distance further than 3,000 bp from an ORF and was excluded from further analysis. Based on the *F. oxysporum *genome map [39], the distribution of the T-DNA integration sites of the pathogenicity mutants was determined and no clustering of the potential pathogenicity genes on the chromosomes was observed (data not shown).

In total, 111 genes potentially involved in pathogenicity were identified (Additional data files 1-3). For most genes, homologues in other fungi were identified; only four putative genes were unique to *F. oxysporum*. Further analysis revealed that in two of these cases a homologue is present in the closely related fungus *F. verticillioides*, which was overlooked in earlier searches due to incorrect annotation of these genes in the *F. oxysporum *genome. The remaining two are small ORFs (100-170 codons) and at present it is not clear whether these are expressed.

For all putative pathogenicity genes a presumed function for the corresponding protein was deducted based on blast searches in combination with functional assignment according to MIPS [40] (Additional data files 1-3). Examples of identified genes and their possible roles in pathogenesis are listed below.

Three known *F. oxysporum *pathogenicity genes were identified: the class V chitin synthase gene *chsV*, the carbon catabolite derepressing protein kinase gene *SNF1*, and the Zn(II)2Cys6 transcription factor gene *FOW2 *[13,25,29]. The class V chitin synthase gene and *FOW2 *were identified more than once (Additional data files 1-3). The reduced or non-pathogenic phenotype of the mutants containing a T-DNA insertion in *SNF1 *or *CHSV *correlated well with the published pathogenicity phenotype of the gene disruption/deletion strains [13,25]. In contrast to the non-pathogenic phenotype described for the *F. oxysporum *f. sp. *melonis FOW2 *deletion mutant [29], two independent insertional mutants identified in this study showed a reduced pathogenicity phenotype. This could be due to residual activity of *FOW2 *in these two mutants due to the location of the T-DNAs, 447 and 690 bp upstream of the *FOW2 *start codon.

In total, 23 proteins were categorized as having a putative role in primary or secondary metabolism, such as metabolism of amino acids, lipids, vitamin B6 or degradation of aromatic compounds. One of these showed high homology to mannose-6-phosphate isomerase, a protein involved in mannose synthesis and a known pathogenicity factor in *Cryptococcus neoformans *[41]. Several genes were found with a potential link to degradation of plant material, for example, those encoding L-threo-3-deoxy-hexulosonate aldolase, an enzyme involved in catabolism of D-galacturonate, a principal component of pectin [42], and catechol dioxygenase and 3-carboxy-cis,cis-muconate cyclase, both involved in metabolism of low-molecular weight aromatic compounds, such as protocatechuate and catechol, degradation products of lignin [43-46]. Also in this class, succinate-semialdehyde dehydrogenase [NADP^+^], an enzyme involved in the GABA-shunt and found to be up-regulated in *F. graminearum *when grown on hop cell wall [47], was identified as a potential pathogenicity factor. In the categories biogenesis of cellular components, protein fate (folding, modification, destination), and cellular transport, transport facilities and transport routes, several proteins belonging to the same biological process were identified. Genes for four different peroxisome biogenesis proteins (Pex1, Pex10, Pex12, and Pex26) were identified. Peroxisomal metabolism has been shown to be important for pathogenicity of *M. grisea *and *Colletotrichum lagenarium *[48,49]. Furthermore, three mutants with a T-DNA insertion in a 20S/26S proteasome subunit and three mutants with a T-DNA insertion in components of the Sec61 protein translocation complex (*SEC61β*, *SEC61α*, and *SEC62*) were found, suggesting an important role for protein translocation and degradation in pathogenesis. Three mutants with insertions in or close to genes belonging to the category cell rescue, defense and virulence were identified; these encode a manganese superoxide dismutase (MnSOD), a putative toxin biosynthesis protein and a RTA1 like protein, which confers resistance to aminocholesterols in *Saccharomyces cerevisiae *[50]. MnSODs have been shown to play a role in pathogenesis in *C. neoformans *and *C. bacillisporus *[51,52]. However, deletion of the *MnSOD *gene had no effect on pathogenicity of *Colletotrichum graminicola *and *Candida albicans *[53,54]. The putative toxin biosynthesis protein shows homology to the product of the host-specific AK-toxin gene *AKT2 *of *Alternaria alternata *(1E-16, 26% identity). Deletion of this gene in *A. alternata *abolished the production of AK-toxin and pathogenicity [55]. In the category development, a gene with homology to the developmental regulator *flbA*, a regulator of G protein signaling (RGS), was identified. RGS proteins accelerate the rate of GTP hydrolysis by Gα proteins and have been shown to play a role in pathogenicity in *C. neoformans*, *Cryphonectria parasitica *and *Metarhizium anisopliae *[56-58]. Finally, scattered over the remaining categories, genes with roles in ion homeostasis, redox balance, ion/multidrug/toxin transport and transcriptional regulation were identified. Ion homeostasis (P-type ATPase), redox balance (NADH-ubiquinone oxidoreductase) and major facilitator superfamily (MFS)/ATP-binding cassette (ABC) transporters have been shown to be important for pathogenesis in various fungi [59].

### Confirmation of a role of peroxisome and cell wall biogenesis genes in pathogenicity through complementation

For three pathogenicity mutants a complementation study was performed to assess whether the pathogenicity phenotype was indeed due to the T-DNA insertion. These are pathogenicity mutants 35F4 (class 2), 83A1 (class 3) and 51D10 (class 3), which are (severely) reduced in pathogenicity (Additional data file 1). Mutant 35F4 contains a single T-DNA insertion in the first exon of *FOXG_02084*, which encodes a protein similar to Peroxin26 (hereafter FoPex26). Reminiscent of other Peroxin26 proteins, which are carboxy-terminally anchored integral peroxisomal membrane proteins [60], FoPex26 also contains a transmembrane region located near the carboxyl terminus (411-433 amino acids) [61,62]. Mutant 83A1 contains two T-DNAs integrated in close proximity to each other. One RB was isolated using TAIL-PCR and was found to be integrated in the ORF of gene *FOXG_08300*, which encodes a protein similar to Peroxin12 (hereafter FoPex12). Similar to other Peroxin12 proteins, FoPex12 contains a pex2/pex12 amino-terminal region (pfam04757) and a carboxy-terminal RING finger domain (pfam00097). Both *pex *mutants grew normally on rich medium (PDA), but were severely reduced in growth on minimal medium (CDA), possibly due to lack of amino acids and vitamins, and, as expected for peroxisomal biogenesis mutants, on medium containing fatty acids as sole carbon source (Additional data file 5). Mutant 51D10 contains two T-DNA insertions located in close proximity (2.2 kb) to each other with one T-DNA being truncated. Two RB flanks were isolated with TAIL-PCR. One RB had integrated 801 bp upstream of *FOXG_05014*, which encodes a conserved hypothetical protein. The second RB had integrated into *FOXG_05013*, which encodes a protein with homology to *Saccharomyces cerevisiae *Dcw1p. *FOXG_05013 *is up-regulated in *F. oxysporum *f. sp. *vasinfectum *during infection of cotton [63]. Therefore, this gene was selected for complementation of the mutant phenotype. Similar to Dcw1p, the protein encoded by *FOXG_05013 *(hereafter FoDcw1) is putatively glycosylphosphatidylinositol (GPI)-anchored and belongs to the family of alpha-1,6-mannanases (glycosyl hydrolase family 76, pfam03663) [61,62]. The pathogenicity mutant displayed no growth abnormalities when grown on various carbon sources.

**Figure 3:**
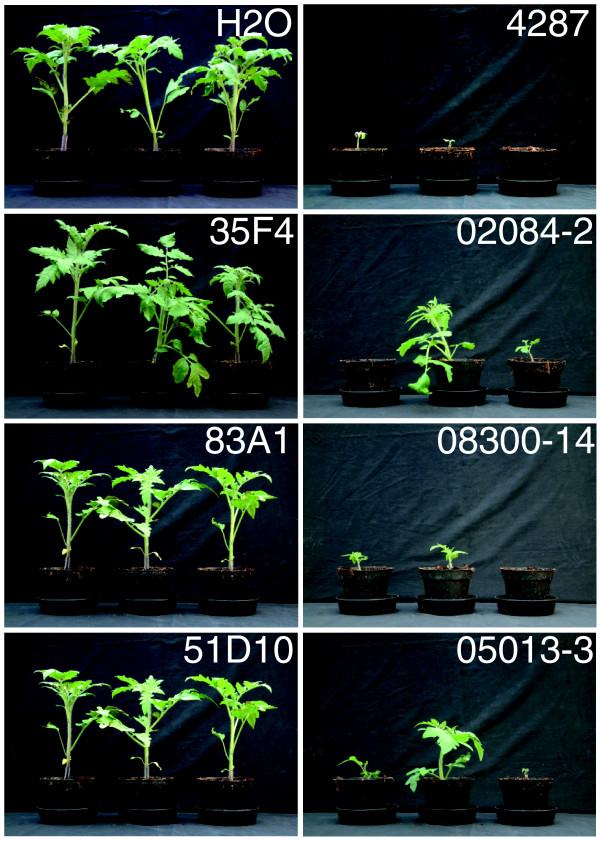
**Peroxisomal biogenesis proteins Pex12 and Pex26 and cell wall protein Dcw1 are necessary for full pathogenicity**. Plant phenotypes three weeks after mock inoculation (H_2_O) or inoculation with *F. oxysporum *wild type 4287, pathogenicity mutant 35F4, 35F4 complementation transformant 02084-2, pathogenicity mutant 83A1, 83A1 complementation transformant 08300-14, pathogenicity mutant 51D10, and 51D10 complementation transformant 05013-3.

**Figure 4:**
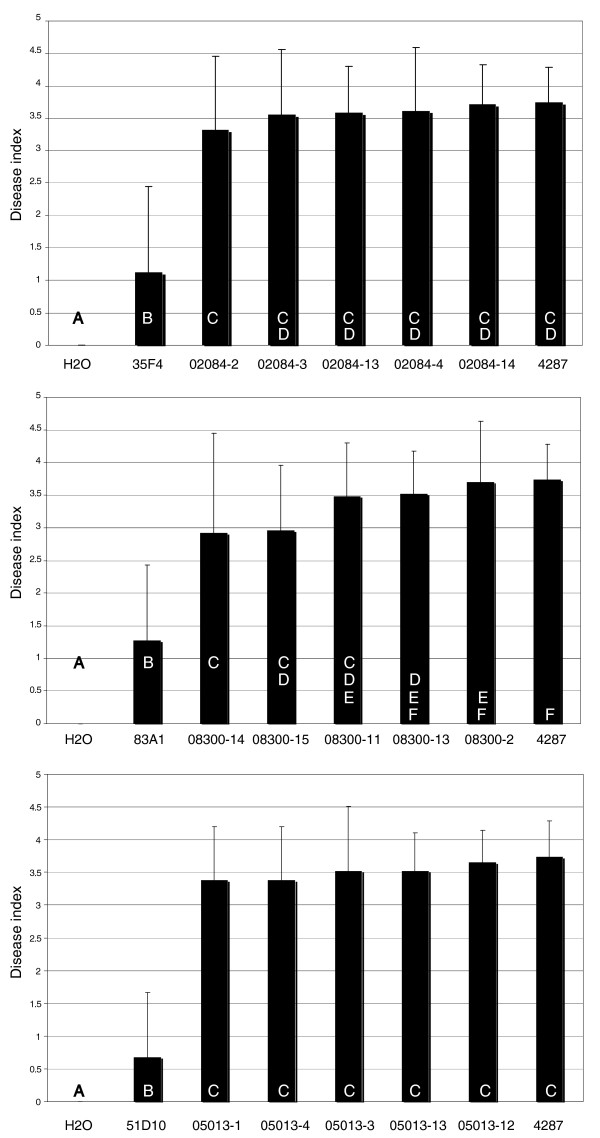
**Peroxisomal biogenesis proteins Pex12 and Pex26 and cell wall protein Dcw1 are necessary for full pathogenicity**. Average disease index of 20 plants three weeks after mock inoculation (H_2_O) or inoculation with *F. oxysporum *wild type 4287, pathogenicity mutants 35F4, 83A1, 51D10 and their complemented counterparts 02084-2 to -14, 08300-2 to -15, and 05013-1 to -13, respectively. Error bars indicate standard deviation and capital letters define statistically different groups (ANOVA, *p *= 0.95).

For FoPex26, FoPex12 and FoDcw1 complementation constructs were generated containing the complete ORF, 650-1,000 bp upstream and 500 bp downstream sequences. These complementation constructs were introduced in the corresponding pathogenicity mutants through *Agrobacterium*-mediated transformation and ectopic integration of the complementation construct was verified by PCR (Additional data file 6). For all three pathogenicity mutants, introduction of an intact copy of the corresponding gene restored the disease causing capacity in five independent transformants (Figure 3). Disease index levels of the complementation mutants were comparable to the disease index of the wild-type infection and in all cases significantly different from the disease index of the corresponding pathogenicity mutant (Figure 4). In addition, the reduced growth phenotype of the Peroxin mutants 35F4 and 83A1 on CDA and fatty acids was complemented in the transformants (Additional data file 5). In conclusion, complementation confirmed that the observed pathogenicity defect in the pathogenicity mutants analyzed was due to the disruption of *FOXG_02084*, *FOXG_08300 *and *FOXG_05013 *and that the proteins FoPex26, FoPex12 and FoDcw1 play a crucial role during infection of tomato by *F. oxysporum*.

### Gene replacement confirms a role in pathogenesis for two out of four genes analyzed

To obtain additional information on the link between T-DNA insertions and phenotypes, we decided to perform a gene knock-out study for four potential pathogenicity genes. The first mutant chosen was 18C4 (disease index <1). This mutant carries a single T-DNA insertion and both the LB and RB were isolated by TAIL-PCR. Analysis revealed that a deletion of seven nucleotides occurred at the insertion point and that the T-DNA was inserted 600 bp upstream of *FOXG_08602*. This gene encodes a protein of 242 amino acids, showing homology to spherulin, a protein thought to be involved in tissue desiccation or hydration [64]. The second mutant, 46D7 (disease index approximately 2), carries a single T-DNA with a truncation of the left border. As a result, only the RB was isolated by TAIL-PCR and analysis revealed that it was inserted 18 bp upstream of *FOXG_03318*. This gene encodes a protein of 586 amino acids with homology to transcriptional regulator Cti6. Like other Cti6 proteins, the protein encoded by *FOXG_03318 *(hereafter FoCti6) contains a PHD finger motif (pfam00628) and a nuclear localization signal (RRRKR at amino acid 68) [61,62]. The third mutant, 54E6 (disease index approximately 1), contains a single T-DNA and, based on the isolated LB and RB in the TAIL-PCR, a 19 nucleotide deletion at the insertion point. The T-DNA was inserted in *FOXG_09487*, which encodes a hypothetical protein with no known domains. Finally, the fourth mutant chosen, 86A9 (disease index <1), contains two T-DNAs integrated in close proximity (approximately 600 bp) of each other. This mutant also contains a chromosomal rearrangement. Three borders were isolated by TAIL-PCR: one RB was inserted 322 bp upstream of *FOXG_09637*, and two LBs were inserted 133 bp upstream and into *FOXG_02054*, respectively. *FOXG_09637 *encodes a hypothetical protein of 387 amino acids with no known domains. *FOXG_02054 *encodes a hypothetical protein of 156 amino acids containing a DUF1183 domain of unknown function (pfam06682) and an amino-terminal signal peptide (amino acids 1-18) [62]. *FOXG_02054 *was chosen as a target for the gene knock out study since it contains a T-DNA insertion in the ORF and is, therefore, the more likely candidate in this mutant.

**Figure 5:**
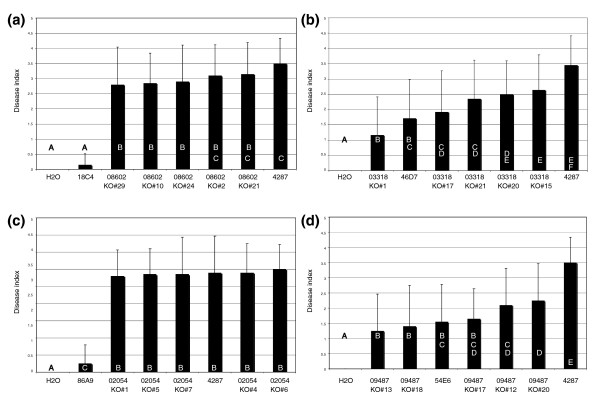
**Transcription factor Cti6 and FOXG_09487 are required for full pathogenicity, whereas FOXG_08602 and FOXG_02054 are not**. **(a-d) **Average disease index of 20 plants three weeks after mock inoculation (H_2_O) or inoculation with *F. oxysporum *wild type 4287, pathogenicity mutant 18C4 and *FOXG_08602 *deletion strains KO#2, 10, 21, 24, and 29 (a); mutant 46D7 and *FOXG_03318 *deletion strains KO#1, 15, 17, 20, and 21 (b); mutant 54E6 and *FOXG_09487 *deletion strains KO#12, 13, 17, 18, and 20 (c); and mutant 86A9 and *FOXG_02054 *deletion strains KO#1, 4, 5, 6, and 7 (d). Error bars indicate standard deviation and capital letters define statistically different groups (ANOVA, *p *= 0.95).

For all four genes, a gene replacement construct was introduced into the wild-type strain and transformants that had undergone homologous recombination were identified by PCR (Additional data files 7-10). Five independent deletion mutants for each gene were assessed in a root dip bioassay to determine their pathogenicity phenotype. Deletion of *FOXG_08602 *and *FOXG_02054 *did not have an effect on pathogenicity. The deletion mutants displayed a disease index that was significantly higher than the corresponding insertional mutagenesis strain and was similar to the disease index obtained with the wild-type strain (Figure 5a,c). In contrast, we were able to confirm a role of *FOXG_03318 *and *FOXG_09487 *in pathogenicity. Deletion of either of these genes led to reduced pathogenicity (Figure 5b,d). Although the deletion mutants belong to statistically different groups, they were all significantly different from the wild-type strain (Figure 5b,d). Some of the apparent differences in pathogenicity between the different deletion mutants may be due to randomly introduced, minor (epi)genetic differences. However, a mutation or an epigenetic modification in the culture used for transformation influencing our results can be excluded since the cultures we used for transformations were not derived from a single spore but were derived from the same mass of mycelium as the wild type control. Therefore, selection of a random mutation affecting pathogenicity of all transformants but not the wild-type control is essentially excluded. In addition, the reduced phenotype in disease causing ability between the mutants and the wild type is reproducible. In conclusion, two lines of evidence support the notion that the tagged genes are involved in pathogenicity: first, the isolation of the original insertional mutant carrying a mutation in or close to the ORF of this gene; and second, the verification of the pathogenicity phenotype in five gene deletion transformants generated independently. Thus, FoCti6 and hypothetical protein FOXG_09487 are required for full pathogenicity of *F. oxysporum *towards tomato.

## Discussion

*Agrobacterium*-mediated transformation is a well established tool for insertional mutagenesis in plants, such as *Arabidopsis thaliana *and *Oryza sativa *[65-67]. When it was demonstrated that this transformation system could also be used for the introduction of DNA into yeast and filamentous fungi [68,69], a new possibility for insertional mutagenesis, besides restriction enzyme mediated insertion (REMI) and transposon-based methodologies, became available as a tool for large-scale insertional mutagenesis in fungi. *Agrobacterium*-mediated insertional mutagenesis has since been successfully used to identify pathogenicity factors in several plant-pathogenic fungi [36-38,70,71]. In this study, *Agrobacterium*-mediated insertional mutagenesis was used to generate pathogenicity mutants of *F. oxysporum *f. sp. *lycopersici *to identify novel genes required for pathogenesis. In total, 10,290 transformants were generated and assessed for loss of pathogenicity on *Solanum lycopersicon *(tomato). Out of these, 106 tranformants (1%) displayed a reproducible non-pathogenic or (severely) reduced pathogenicity phenotype and 111 potential pathogenicity genes were identified. Assuming a genome size of 61 Mb, 17,735 genes [39], and random T-DNA insertion events, and taking into consideration that several variables are unknown (such as the exact number and length of transcription units, the average distance to an ORF for an insertion to affect gene expression, the number of essential genes and the role of chromatin state (gene activity) on the likelihood of T-DNA insertion), we estimate a genome coverage of 30-40%. Indeed, several confirmed or potential pathogenicity genes were identified more than once, such as those encoding chitin synthase V (three times), developmental regulator FlbA (twice), a hypothetical protein with homology to Ryp1 (three times), carboxy-cis,cis,muconate cyclase (twice) and transcription factor FOW2 (twice), indicating that some redundancy is present in this collection of transformants. However, most known *F. oxysporum *pathogenicity genes (14 to18 out of the 20 known genes would have been possible to identify in our screen; see Introduction) were not identified in our screen, which underlines the incompleteness of our collection. Our estimations of the average T-DNA copy number, the percentages of single T-DNA integrations, truncations of the LB and RB, non-T-DNA insertions and chromosomal rearrangements and deletions of more than 1 kb are similar to what has been observed in insertional mutant collections generated with *A. tumefaciens *in other fungi, as well as in plants [36-38,65,67,72-77]. This indicates that the process of T-DNA integration into the genome of *F. oxysporum *is essentially similar to that observed in other organisms. Taken together, we conclude that *Agrobacterium*-mediated insertional mutagenesis is well suited for creating *F. oxysporum *mutants with desired phenotype(s).

In this study, only when a T-DNA was inserted in an ORF, or within 1 kb up- or downstream of an ORF, was it assumed that expression of the gene could be influenced by the T-DNA insertion and the gene was marked as a potential pathogenicity gene. This resulted in the identification of 111 potential pathogenicity genes (Additional data files 1-3). Among these, several encode known pathogenicity factors, underscoring the effectiveness of *Agrobacterium*-mediated insertional mutagenesis to identify pathogenicity factors in *F. oxysporum*. However, from other insertional mutagenesis screens, it is known that not every T-DNA insertion is linked to the mutant phenotype. For *C. neoformans *and *M. grisea *the percentage of tagged mutants ranged from 53% to 93% [38,70,78]. Therefore, it should be kept in mind that some of the genes identified might not be involved in pathogenicity, as demonstrated by deletion of *FOXG_08602 *and *FOXG_02054*. Nevertheless, functional categorization of the entire list of genes indicated that certain metabolic pathways (amino acid and lipid metabolism), cell wall integrity, and certain cellular processes (protein translocation and degradation) appear to be important for full pathogenicity of *F. oxysporum*. A striking observation was that mutants impaired in primary metabolism or mutants with a severe growth phenotype on CDA (the selective medium) were identified. We hypothesize that although the selection medium does not support auxotrophic growth, these mutants were still able to grow due to the presence of non-transformed fungal hyphae and *A. tumefaciens *cells, which might have served as a nutrient supply. Comparison of our list of potential pathogenicity genes to 672 potential pathogenicity genes published for *M. grisea *[79] revealed five common (that is, orthologous) genes. Only those T-DNA insertions located within 1,000 bp of an ORF were included in the comparison. The common genes were the known pathogenicity genes for chitin synthase V (*chsV*), a GTPase activating protein (*FOXG_07699*), a RNA polymerase II transcription mediator (*FOXG_08531*), a pyridoxine biosynthesis protein involved in vitamin B6 metabolism (*FOXG_08652*) and a Ca^2+ ^permease/membrane transporter involved in calcium homeostasis (*FOXG_11097*). The fact that these genes were found in insertional mutagenesis screens in two plant-pathogenic fungi points strongly towards a conserved requirement of these proteins for colonization of living plants.

The link between T-DNA insertion and pathogenicity phenotype was confirmed for five of seven genes tested either by complementation or gene knock-out. Complementation of two pathogenicity mutants (Pex12 and Pex26) with the corresponding genes completely restored the capacity to cause disease on tomato seedlings, indicating that peroxisomal function is important for pathogenicity of *F. oxysporum*. Peroxisomes are single membrane bound organelles possessing multiple metabolic functions in filamentous fungi, including β-oxidation of fatty acids, peroxide detoxification and occlusion of septal pores [80-82]. More than 20 peroxin proteins involved in peroxisome biogenesis have been identified in fungi [60]. Mutations in peroxin genes usually result in the absence of normal peroxisomes, mislocalization of peroxisomal matrix proteins and inability to perform specific biochemical reactions, such as fatty acid catabolism [60]. Four different pex genes, *PEX1*, *PEX10*, *PEX12*, and *PEX26*, were identified as potential pathogenicity genes in this study. The four peroxins encoded by these genes are all involved in docking and translocation of receptor-cargo moieties across the peroxisomal membrane [60,83]. It can therefore be assumed that inactivation of any one of these peroxins disturbs peroxisomal function, as shown by the reduced growth of these mutants on fatty acids. Peroxisomal function and fatty acid metabolism have been shown to play a role during fungal pathogenesis in *C. lagenarium*, *M. grisea*, and *Ustilago maydis *[38,48,49,84,85]. Inactivation of *PEX6 *in *C. lagenarium *and *M. grisea *resulted in disturbed peroxisomal function, formation of aberrant appressoria, and failure to penetrate the host cuticle [48,49,85]. Failure to penetrate the host cuticle was attributed to the aberrant appressoria, which were smaller in size and severely reduced in melanization, and to the absence of appressorium lipolysis [48,49,85]. We here show that peroxisomal function is also necessary for pathogenesis of a root infecting fungus that lacks appressoria. It could still be that peroxisomal function is necessary for efficient root penetration. Given the incomplete loss of pathogenicity, the insertion mutants would then penetrate the root cortex at a reduced efficiency. Another possibility is that peroxisomal function is necessary for proper utilization of host nutrients during *in planta *growth as has been postulated for *U. maydis *[84]. A third possibility is that *F. oxysporum pex *mutants are more sensitive to cytoplasmic leakage during *in planta *growth due to absence of woronin bodies. Woronin bodies have been identified as a special class of peroxisomes and disruption of peroxisomal function also disturbs the biogenesis of woronin bodies [80,81,86]. Combined loss of peroxisomal function and woronin bodies has been observed in a *M. grisea PEX6 *deletion mutant [49].

Another process that has been shown to be important for pathogenicity of *F. oxysporum *is maintenance of cell wall integrity [12-16]. We also identified genes with a function in cell wall assembly as potential pathogenicity genes, such as those encoding chitin synthase V, mannose-6-phosphate isomerase and a GPI-anchored protein with homology to *S. cerevisiae *Dcw1p. For the first two proteins it was demonstrated earlier that their function is required for pathogenesis and/or for maintaining cell wall integrity in various filamentous fungi [13,41,87,88]. We show here that introduction of the *F. oxysporum DCW1 *gene into the *dcw1 *pathogenicity mutant restores pathogenicity, thereby confirming that FoDcw1 is also necessary for pathogenesis. In *S. cerevisiae*, the absence of Dcw1p leads to defects in bud growth and cell polarity. In addition, an aberrant cell wall with increased chitin content was observed and it was hypothesized that Dcw1p is necessary for normal biogenesis of the cell wall [89,90]. It could be that disturbance of FoDcw1 function in *F. oxysporum *also leads to a weakened cell wall. This in turn could lead to less efficient penetration of the root cortex, reduced *in planta *growth and/or an increased sensitivity to plant defense mechanisms. Alternatively, it could be that an altered cell wall induces host defense responses through exposure or release of molecular patterns that are recognized by the host. The *dcw1 *mutant was not disturbed in superficial root colonization and was not more sensitive to the cell wall disturbing reagents glucanex and SDS or to high concentrations of sorbitol. However, a minor swelling of the hyphae was observed when the mutant was grown in the presence of calcofluor white (data not shown), indicating that cell wall integrity may be disturbed to some extent in this mutant. Overall, *FoDCW1 *is not required for normal development, since no effect on growth on various carbon sources or on sporulation was observed.

Another gene verified here to be important for *F. oxysporum *pathogenicity is *FOXG_09487*, which encodes a conserved hypothetical protein. Further studies should be performed to determine what role this protein plays during pathogenicity. Finally, we were able to verify that *FoCTI6 *is required for full pathogenicity. Its product, FoCti6, is likely involved in transcriptional regulation based on homology to *S. cerevisiae *Cti6. Papamichos-Chronakis *et al. *[91] studied the role of Cti6 in transcriptional activation and repression in *S. cerevisiae *by using the *GAL1 *promoter as a model and they showed that Cti6 simultaneously interacts with the transcriptional co-repressor complex Cyc8-Tup1 and the co-activator SAGA (Spt-Ada-Gcn5-acetytransferase) complex. Co-repressor complex Cyc8-Tup1 controls many physiological pathways, such as glucose repression, hypoxia, and cell type specific expression [92]. The SAGA complex functions as a co-activator complex through histone-post-translational modification and gene regulation [93]. Interestingly, one of the *F. oxysporum *pathogenicity mutants identified in this study has completely lost *FOXG_08594*. The product of this gene shows high homology to one of the components of the SAGA complex, Spt3. This protein has been shown to be required for *C. albicans *virulence and to play opposite roles in filamentous growth in *S. cerevisiae *and *C. albicans *[94]. We hypothesize that in *F. oxysporum*, certain genes required for pathogenesis are under transcriptional regulation of FoCti6 and possibly the SAGA complex.

## Conclusion

Large scale *Agrobacterium*-mediated insertional mutagenesis was used successfully to identify novel pathogenicity genes in *F. oxysporum*. About one in a hundred transformants was reduced in pathogenicity and 111 potential pathogenicity genes were identified. Functional categorization of the potential pathogenicity genes indicate that certain metabolic pathways, cell wall integrity, and a subset of cellular processes seem to be important for full pathogenicity of *F. oxysporum *on tomato. In addition, knock-out and complementation studies confirmed that a GPI-anchored protein thought to be involved in cell wall integrity, a transcriptional regulator, a protein with unknown function and peroxisomal function are required for full pathogenicity. Verification and characterization of additional potential pathogenicity genes is in progress. Together, these efforts should lead to a comprehensive picture of the molecular requirements for pathogenicity of a root-invading fungus.

## Materials and methods

### Strains, plant material, media, culture conditions

*F. oxysporum *f. sp. *lycopersici *strain 4287 (race 2), used as the parent strain for fungal transformation, was obtained from A Di Pietro, Universidad de Córdoba, Spain. It was stored as a monoconidial culture at -80°C and revitalized on potato dextrose agar (PDA, Difco, Le Pont de Claix, France) at 25°C. *Agrobacterium tumefaciens *EHA105 [95] was used for *Agrobacterium*-mediated transformation of *F. oxysporum *and was grown in Luria broth medium [96] containing 20 μg/ml rifampicin at 28°C. Introduction of the plasmids into the *Agrobacterium *strain was performed as described by Mattanovich *et al*. [97]. *Escherichia coli *DH5 alpha (Invitrogen, Breda, The Netherlands) was used for construction, propagation, and amplification of the plasmids and was grown in Luria broth medium at 37°C containing either 100 μg/ml ampicillin or 50 μg/ml kanamycin depending on the resistance marker of the plasmid used. Plant line Moneymaker ss590 (Gebr. Eveleens b.v., Aalsmeer, The Netherlands) was used to assess pathogenicity of *F. oxysporum *strains and transformants.

### Construction of insertional mutagenesis transformation vector

The insertional mutagenesis transformation vector pPK2*hphgfp *contains between the left and right T-DNA border an in-frame fusion of the hygromycin resistance gene with green fluorescent protein (GFP) under the control of the *Aspergillus nidulans *glyceraldehyde-3-phosphate dehydrogenase (*gpdA*) promoter and *A. nidulans trpC *terminator. pPK2*hphgfp *was constructed in a two-step approach. First, plasmid pPK2 [98] was amplified entirely by PCR using primers HPH-Fwd2-ApaI and HPH-End-ApaI-f (Additional data file 11) in order to introduce an *Apa*I restriction site before the stop codon of the *hph *gene. The pPK2ApaI PCR fragment was digested with *Apa*I and ligated, resulting in plasmid pPK2*hphApa*I. The *gfp *coding sequence was amplified by PCR using primers GFP-f-ApaI and GFP-r-ApaI (Additional data file 11) with plasmid pGPDGFP [99] as a template. The *gfp *PCR fragment was digested with *Apa*I and cloned into pPK2*hphApa*I previously linearized with *Apa*I, resulting in pPK2*hphgfp*.

### Construction of complementation constructs

Three complementation constructs containing the ORFs of *FOXG_05013*, *FOXG_02084*, and *FOXG_08300 *and sequences of several hundred base-pairs up- and downstream of them were constructed. Using *pfu *polymerase (Fermentas, St. Leon-Rot, Germany) and the primer pairs FOXG_05013f-XbaI/FOXG_05013r-AscI, FOXG_02084f-AscI/FOXG_02084r-XbaI and FOXG_08300f-XbaI/FOXG_08300r-AscI (Additional data file 11), PCR products were generated corresponding to the ORF of *FOXG_05013 *together with sequence 740 bp upstream and 500 bp downstream of it, to the ORF of *FOXG_02084 *together with sequence 649 bp upstream and 500 bp downstream of it, and to the ORF of *FOXG_08300 *together with sequence 1,000 bp upstream and 500 bp downstream of it, respectively. The PCR products were cloned into pGEM-T Easy (Promega, Leiden, The Netherlands) and sequenced on an ABI PRISM genetic analyzer using an ABI PRISM BigDye Terminator kit (Applied Biosystems, Nieuwekerk a/d IJssel, The Netherlands) and the corresponding FOXG primers listed in Additional data file 11. Subsequently, pGEMtFOXG_02084, pGEMtFOXG_05013 and pGEMtFOXG_08300 were digested with *Xba*I and *Asc*I and the FOXG *Xba*I/*Asc*I fragments were cloned into *Xba*I/*Asc*I-digested pRW1p [100], resulting in pRW1pFOXG_02084, pRW1pFOXG_05013 and pRW1pFOXG_08300, respectively.

### Construction of gene disruption constructs

Gene disruption constructs containing a hygromycin resistance cassette and sequences of *FOXG_08602*, *FOXG_03318*, *FOXG_09487 *and *FOXG_02054 *including several base-pairs upstream and downstream of them were generated. PCR products corresponding to the upstream and downstream sequences were generated using *pfu *polymerase (Fermentas) and the primer pairs FOXG_08602-f3/FOXG_08602-r3 and FOXG_08602-f4/FOXG_08602-r4, FOXG_03318-f1/FOXG_03318-r1 and FOXG_03318-f2/FOXG_03318-r2, FOXG_09487-f1/FOXG_09487-r1 and FOXG_09487-f2/FOXG_09487-r2 and 86A9pro_fw/86A9pro_rev and 86A9term_fw/86A9term_rev (Additional data file 11). The PCR products were cloned into pGEM-T Easy (Promega) and sequenced on an ABI PRISM genetic analyzer using ABI PRISM BigDye Terminator kit (Applied Biosystems), using M13forward and M13reverse primers for sequencing. Subsequently, an *Asc*I/*Hind*III- or *Kpn*I/*Pac*I-digested fragment corresponding to the *FOXG_08602 *promoter and terminator region, a *Kpn*I/*Pac*I- or *Hind*III/*Xba*I-digested fragment corresponding to the *FOXG_03318 *terminator or promoter region, a *Kpn*I/*Pac*I- or *Hind*III/*Xba*I-digested fragment corresponding to the *FOXG_09487 *terminator or promoter region, or a *Kpn*I/*Pac*I- or *Asc*I/*Xba*I-digested fragment corresponding to the *FOXG_02054 *terminator or promoter region were sequentially cloned in pRW2h [100], resulting in pRW2hFOXG_08602KO, pRW2hFOXG_03318KO, pRW2hFOXG_09487KO or pRW2hFOXG_02054KO, respectively.

### Fungal transformation, pathogenicity and growth assays

*Agrobacterium*-mediated transformation of *F. oxysporum *f. sp. *lycopersici *was performed as described by Mullins *et al*. [101] with minor adjustments [102]. Depending on the selection marker used, transformants were selected on Czapek Dox agar (CDA, Oxoid, Basingstoke, Hampshire, England) containing 100 μg/ml Hygromycin (Duchefa, Haarlem, The Netherlands) or on CDA containing 0.1 M TrisHCl pH 8 and 100 μg/ml Zeocin (InvivoGen, Toulouse, France).

Plant infection was performed using 9- to 11-day-old seedlings (Moneymaker ss590) and following the root-dip inoculation method [103]. For the high throughput three-plants-in-one-pot bioassays, the inoculum was prepared by growing the independent *F. oxysporum *transformants on PDA medium for 5 to 6 days followed by collection of microconidia using 5 ml of sterilized water. The conidial suspensions were collected in 24-well plates (one transformant per well) and in each well three uprooted tomato seedlings were incubated for about one minute. Subsequently, the three seedlings were planted in one container (12 cm in diameter) containing potting soil. After three weeks the degree of infection was determined by visual inspection.

For the standard bioassay the inoculum was prepared by growing *F. oxysporum *for 5 days in NO_3 _medium (0.15% Yeast Nitrogen Base, 3% sucrose, 100 mM KNO_3_) at 25°C and shaking at 150 rpm. Cultures were then filtered through one layer of Miracloth (Calbiochem, Darmstadt, Germany), centrifuged and washed with sterile water, and adjusted to a concentration of 10^7 ^conidia/ml. The uprooted seedlings (20 per fungal strain) were incubated for about one minute in the conidial suspension and individually planted in a container (12 cm in diameter) containing potting soil. The inoculated plants were grown in a random block design (five replicates per block). After three weeks, the degree of vascular browning and the weight above the cotyledons were determined for each plant. Disease index was scored on a scale from 0 to 4 (0, healthy plant; 1, slightly swollen hypocotyl; 2, one brown vascular bundle in hypocotyl; 3, two or three brown vascular bundles and/or severe growth distortion (small plant and/or asymmetric development); 4, more than three brown vascular bundles and/or very small wilted plant or dead). Statistical analysis (ANOVA and Fisher *post hoc *test) was performed using StatView™SE+ v1.03.

All transformants tested in the standard bioassay were also subjected to growth tests. For this purpose 2 μl of the conidial suspension (10^7 ^conidia/ml) was spotted on CDA, PDA, and minimal medium (0.17% Yeast Nitrogen Base, 50 mM KNO_3 _and 1.5% bacto agar (Difco)) supplemented either with 1% ethanol, glycerol, malic acid pH 6.0, citric acid pH 6.0, sucrose or no carbon source. After an incubation period of 7 days at 25°C the transformants were scored based on colony diameter and growth phenotype in comparison to the wild type strain. The growth assay of the *pex *mutants on minimal medium containing 1% Tween20, oleic acid or olive oil was performed as described above.

### Isolation of T-DNA flanking regions

TAIL-PCR was used to isolate genomic DNA flanking the inserted T-DNA from those transformants that were significantly reduced in pathogenicity. The TAIL-PCR conditions were adapted from the protocol described by Mullins *et al*. [101] and are described in Additional data file 12. The primers for isolating the RB flanking regions and arbitrary degenerate primer AD1 were described by Mullins *et al*. [101]. Primers used for isolating the LB flanking regions and additional degenerate primers used in this study are listed in Additional data file 11. Depending on the degenerate primer used, the final concentration of the primers in the TAIL-PCR were 0.4 μM specific primer and 4 μM degenerate primer for AD1 or AD4 or 0.2 μM specific primer and 1.5 μM degenerate primer for AD2, AD3, AD6, AD7, or AD8. The tertiary TAIL-PCR product(s) of each transformant was/were purified using Qiaquick columns (Qiagen, Hilden, Germany) and ligated into pGEM-T Easy (Promega). Colony PCR was used to identify the correct clones using primers M13forward and M13reverse (Additional data file 11). The PCR products from the colony PCR were sequenced on an ABI PRISM genetic analyzer using ABI PRISM BigDye Terminator kit (Applied Biosystems) and M13forward and M13reverse as sequence primers. After *in silico *removal of T-DNA border sequences, the sequences of the TAIL-PCR product were used to search for nucleotide and amino acid similarities by BLASTN and BLASTX algorithms, respectively, against the *Fusarium *group genome database [39] and NCBI [104].

### Molecular analysis of the transformants

*F. oxysporum *was grown in 50 ml NH_4 _medium (0.17% Yeast Nitrogen Base, 3% sucrose, 50 mM (NH_4_)_2_SO_4_) for 5 days at 25°C shaking at 175 rpm. Mycelium was harvested by filtration through Miracloth, washed with deionized water and freeze-dried overnight. Genomic DNA was isolated as described by Kolar *et al*. [105] with minor adjustments. Briefly, 2 ml DNA-extraction buffer (0.2 M TrisHCL pH 8.5, 0.25 M NaCl, 0.05 M EDTA pH 8.0, 48 mg/ml sodium 4-aminosalicylate dihydrate (PAS, Sigma-Aldrich, Zwijndrecht, The Netherlands), 8 mg/ml Triisopropylnaphthalenesulfonic acid sodium (TIPS, Sigma-Aldrich) and 2 ml water-saturated phenol-chloroform (1:1) was added to ground mycelium, mixed and centrifuged for 30 minutes at 3,500 rpm and 4°C. DNA was precipitated from the aqueous phase with 0.7 volume isopropanol and 0.1 volume 4 M NaCl and centrifugation for 30 minutes at 3,500 rpm and 4°C. The pellet was resuspended in 400 μl TE buffer (10 mM TrisHCL pH7.5, 1 mM EDTA pH 8.0) and extracted three times with water-saturated phenol:chloroform (1:1). DNA was then precipitated with 2 volumes 96% ethanol and 0.1 volume 3 M NaAc. The DNA was finally dissolved in 100 μl deionized water.

For Southern analysis, 10 μg genomic DNA of each transformant was digested with 20 U *Bgl*II or 20 U *Bam*HI overnight at 37°C. The samples were loaded on a 1% 0.5× Tris-borate/EDTA gel and run for 18 h at 45 V. The digested DNA was transferred to Hybond-N+ (Amersham Pharmacia, Diegem, Belgium) as described by Sambrook *et al*. [96]. The probes used for Southern analysis were a 1,744 bp *Nhe*I-*Nco*I and a 780 bp *Bam*HI-*Hind*III fragment of plasmid pPK2*hphgfp *corresponding to the *gpdA *promoter and a part of the hygromycin resistance gene (*hph*) and *trpC *terminator, respectively. Plasmid pPZP201BK [106] was used for detection of the binary backbone. For the detection of the left and right T-DNA border 381 bp and 327 bp PCR products were generated using primers LB-f and pPK2-LB1 and primers pPK2-RB1 and RB-r, respectively [101] (Additional data file 11). The plasmid pPK2*hphgfp *was used as template DNA for this PCR. The DecaLabel™ DNA Labeling Kit (Fermentas) was used to label probes with [α-^32^P]dATP. Hybridization was done overnight at 65°C in 0.5 M sodium phosphate buffer, pH 7.2, containing 7% SDS and 1 mM EDTA. Blots were washed with 0.2 × SSC, 0.1% SDS. Hybridization signals were visualized by phosphorimaging (Molecular Dynamics, Diegem, Belgium).

## Abbreviations

CDA: Czapek-Dox agar; GFP: green fluorescent protein; GPI: glycosylphosphatidylinositol; LB: left border; ORF: open reading frame; PDA: potato dextrose agar; RB: right border; TAIL-PCR: thermal asymmetric interlaced PCR; T-DNA: transfer DNA.

## Authors' contributions

MR and CM designed the study; CM, RvW and LR carried out the experiments and performed data processing; CM interpreted the data and wrote the manuscript; BC provided guidance and review.

## Additional data files

The following additional data are available with the online version of this paper. Additional data file 1 is a table listing pathogenicity mutants with a T-DNA insertion in an ORF. Additional data file 2 is a table listing pathogenicity mutants with a T-DNA insertion within 500 bp up- or 200 bp downstream of an ORF. Additional data file 3 is a table listing pathogenicity mutants with a T-DNA insertion within 1,000-500 bp up- or 200-1,000 bp downstream of an ORF. Additional data file 4 is a table listing pathogenicity mutants with a T-DNA insertion in an 'intergenic' region (defined as 3,000-1,000 bp up- or downstream of an ORF). Additional data file 5 is a figure showing that the growth of the *pex *mutants is disturbed on minimal medium and fatty acids. Additional data file 6 is a figure depicting the method and analysis of transformants complemented with *FOXG_02084*, *FOXG_08300 *or *FOXG_05013*.

Additional data file 7 is a figure depicting the method and analysis of transformants deleted for *FOXG_08602*. Additional data file 8 is a figure depicting the method and analysis of transformants deleted for *FOXG_03318*. Additional data file 9 is a figure depicting the method and analysis of transformants deleted for *FOXG_09487*.

Additional data file 10 is a figure depicting the method and analysis of transformants deleted for *FOXG_02054*. Additional data file 1111 is a table listing primer sequences used for PCR and sequencing. Additional data file 12 is a table listing the conditions used for TAIL-PCR.

## Supplementary Material

Additional data file 1 Pathogenicity mutants with a T-DNA insertion in an ORF. Click here for file

Additional data file 2 Pathogenicity mutants with a T-DNA insertion within 500 bp up- or 200 bp downstream of an ORF. Click here for file

Additional data file 3 Pathogenicity mutants with a T-DNA insertion within 1,000-500 bp up- or 200-1,000 bp downstream of an ORF. Click here for file

Additional data file 4 Iintergenic regions are defined as 3,000-1,000 bp up- or downstream of an ORF. Click here for file

Additional data file 5 Growth of the *pex *mutants is disturbed on minimal medium and fatty acids. Click here for file

Additional data file 6 Method and analysis of transformants complemented with *FOXG_02084*, *FOXG_08300 *or *FOXG_05013*. Click here for file

Additional data file 7 Method and analysis of transformants deleted for *FOXG_08602*. Click here for file

Additional data file 8 Method and analysis of transformants deleted for *FOXG_03318*. Click here for file

Additional data file 9 Method and analysis of transformants deleted for *FOXG_09487*. Click here for file

Additional data file 10 Method and analysis of transformants deleted for *FOXG_02054*. Click here for file

Additional data file 11 Primer sequences used for PCR and sequencing. Click here for file

Additional data file 12 Conditions used for TAIL-PCR. Click here for file
