# Supplementary material for: Insight into the molecular requirements for pathogenicity of Fusarium oxysporum f. sp. lycopersici through large-scale insertional mutagenesis
Source: Genome Biol. 2009 Jan 9;10(1):R4. doi: 10.1186/gb-2009-10-1-r4 (PMC2687792; doi:10.1186/gb-2009-10-1-r4)
Supplement: Additional data file 1 — Pathogenicity mutants with a T-DNA insertion in an ORF. [file gb-2009-10-1-r4-S1.doc]

Table S1. Pathogenicity mutants with a T-DNA insertion in an ORF.

| mutant ID number | growth phenotype^a^ | pathogenicity phenotype | locus | blast hits | | |
| --- | --- | --- | --- | --- | --- | --- |
|  |  | (disease index) |  | description | organism | *E*-value |
| 5G2 | - | 0 | FOXG_10510 | Ryp1 | *Ajellomyces capsulatus* | 2.00E-67 |
| 8H8 | - | 1.6 | EST FVNBS82TH | - | - | - |
| 10B1 | - | 1.4 | FOXG_08562 | MFS multidrug transporter | *Aspergillus fumigatus* | 3.00E-148 |
| 10D1 | +/- | 1.1 | FOXG_03332 | phosphoadenosine phosphosulfate reductase | *Neurospora crassa* | 1.00E-115 |
| 14E3 | +++ | 0 | FOXG_11154 | no significant hits |  |  |
| 17G9 | +/- | 2.7 | FOXG_11061 | no significant hits |  |  |
| 18A1 | +/- | 0.2 | FOXG_11632 | amidase family protein | *Aspergillus clavatus* | 0E |
| 19E11 | - | 0 | FOXG_16711 OR FOXG_16779 | acetyl xylan esterase | *Neosartorya fischeri* | 1.00E-56 |
| 21A3 | +/- | 0.6 | FOXG_01032 | D-lactate dehydrogenase, mitochondrial precursor | *Neurospora crassa* | 0E |
| 30C11 | - | 1.1 | FOXG_06232 | hypothetical protein FG09534.1 | *Gibberella zeae* | 0E |
| 30C11 | - | 1.1 | FOXG_12728 | cAMP-independent regulatory protein pac2 | *Aspergillus terreus* | 5.00E-87 |
| 31E10 | + | 0.6 | FOXG_13757 | manganese superoxide dismutase | *Cordyceps militaris* | 1.00E-94 |
| 32D8 | - | 0.6 | FOXG_08482 | developmental regulator *flbA* | *Neurospora crassa* | 0E |
| 35E9 | - | 0.3 | FOXG_08482 | developmental regulator *flbA* | *Neurospora crassa* | 0E |
| 35F4 | + | 0.6 | FOXG_02084 | peroxin 26 | *Penicillium chrysogenum* | 4.00E-87 |
| 36B3 | ++ | 0 | FOXG_06299 | MFS aflatoxin efflux pump | *Neurospora crassa* | 9.00E-179 |
| 36B3 | ++ | 0 | FOXG_06300 | hypothetical protein FG09596.1 | *Gibberella zeae* | 2.00E-27 |
| 38G9 | +/- | 0 | FOXG_04162 | class V chitin synthase | *Fusarium oxysporum* | 0E |
| 43A7 | +++ | 0 | FOXG_10442 | sulfite reductase beta subunit | *Neurospora crassa* | 0E |
| 44H1 | - | 0.1 | FOXG_03305 | hypothetical protein FG08502.1 | *Gibberella zeae* | 3.00E-132 |
| 46A1 | - | 0.4 | FOXG_05430 | SWR1-complex protein 4 | *Neurospora crassa* | 1.00E-130 |
| 46A1 | - | 0.4 | FOXG_05270 | related to U1 small nuclear ribonucleoprotein C | *Neurospora crassa* | 4.00E-39 |
| 46A1 | - | 0.4 | FOXG_04898 | non ribosomal peptide synthase | *Metarhizium anisopliae* | 7.00E-135 |
| 46D3 | - | 1.7 | FOXG_08597 | kinesin | *Gibberella moniliformis* | 0E |
| 46D9 | - | 1.1 | FOXG_00651 | hypothetical protein FG00402.1 | *Gibberella zeae* | 0E |
| 47F6 | +/- | 0.7 | FOXG_01698 | peroxin-1 | *Penicillium chrysogenum* | 0E |
| 48A10 | - | 0 | FOXG_10511 | GDP-mannose transporter | *Neurospora crassa* | 1.00E-159 |
| 48C2 | - | 1.1 | FOXG_01884 | NADH-ubiquinone oxidoreductase B14 subunit | *Magnaporthe grisea* | 3.00E-56 |
| 49G4 | +/- | 0.6 | FOXG_01730 | RING-1 (peroxin 10) | *Gibberella zeae* | 2.00E-127 |
| 51A12 | - | 0 | FOXG_09857 | RTA1 like protein | *Neosartorya fischeri* | 3.00E-55 |
| 51A12 | - | 0 | FOXG_09855 | catechol dioxygenase | *Neosartorya fischeri* | 1.00E-96 |
| 51A12 | - | 0 | FOXG_10252 | proteasome regulatory particle subunit Rpt5 | *Magnaporthe grisea* | 0E |
| 51D10 | - | 1 | FOXG_05013 | putative DFG5 protein | *Neurospora crassa* | 2.00E-172 |
| 53C4 | +++ | 0.3 | FOXG_05165 | spermidine synthase (putrescine aminopropyltransferase) | *Gibberella zeae* | 2.00E-114 |
| 54E6 | - | 1.1 | FOXG_09487 | hypothetical protein FG05415.1 | *Gibberella zeae* | 3.00E-172 |
| 54E11 | +++ | 0 | FOXG_03226 | hypothetical protein FG08430.1 | *Gibberella zeae* | 0E |
| 54H7 | - | 1.8 | FOXG_10377 | P-type Na+-ATPase | *Fusarium oxysporum* | 0E |
| 56G4 | - | 0.1 | FOXG_04162 | class V chitin synthase | *Fusarium oxysporum* | 0E |
| 62C1 | ++ | 0 | FOXG_03489 | hypothetical protein FG08336.1 | *Gibberella zeae* | 2.00E-122 |
| 63B2 | - | 2.1 | FOXG_02385 | UPF0187 domain membrane protein | *Neosartorya fischeri* | 3.00E-121 |
| 66A3 | + | 0.7 | FOXG_11617 | XPA-binding protein 1 | *Aspergillus terreus* | 2.00E-129 |
| 68G11 | - | 2.2 | FOXG_11129 | hypothetical protein FG01577.1 | *Gibberella zeae* | 9.00E-175 |
| 69B3 | - | 1.9 | FOXG_06056 | DNA damage response protein RcaA | *Neosartorya fischeri* | 6.00E-96 |
| 69C3 | +/- | 0.1 | FOXG_02962 | short-chain dehydrogenase/reductase 2 | *Aspergillus clavatus* | 1.00E-52 |
| 71D5 | - | 1.9 | EST FVOCA89TH | - | - | - |
| 72C7 | +/- | 0 | FOXG_06178 | 3-carboxy-cis,cis-muconate cyclase | *Magnaporthe grisea* | 2.00E-73 |
| 73G10 | +/- | 0.2 | FOXG_13915 | no significant hits |  |  |
| 75H3 | - | 0.1 | FOXG_06178 | 3-carboxy-cis,cis-muconate cyclase | *Magnaporthe grisea* | 2.00E-73 |
| 82F7 | +/- | 0.2 | FOXG_03050 | MFS multidrug transporter | *Aspergillus fumigatus* | 8.00E-60 |
| 83A1 | +/- | 0.9 | FOXG_08300 | peroxisome biosynthesis protein PAS10/Peroxin-12 | *Aspergillus clavatus* | 1.00E-142 |
| 86A9 | + | 0.1 | FOXG_02054 | DUF1183 domain protein | *Aspergillus clavatus* | 2.00E-34 |
| 88E1 | +/- | 1.0 | FOXG_08405 | hypothetical protein FG09664.1 | *Gibberella zeae* | 0E |
| 88E1 | +/- | 1.0 | FOXG_07699 | TBC domain protein | *Aspergillus fumigatus* | 3.00E-113 |
| 88E1 | +/- | 1.0 | FOXG_11230 | arrestin domain protein | *Aspergillus clavatus* | 3.00E-163 |
| 89A6 | +/- | 0.1 | FOXG_05528 | protein kinase SNF1 | *Fusarium oxysporum* | 0E |
| 89F6 | - | 0.6 | FOXG_03001 | toxin biosynthesis protein | *Aspergillus fumigatus* | 2.00E-105 |
| 90C8 | +/- | 0.7 | FOXG_11185 | translocon protein Sec61beta | *Aspergillus clavatus* | 2.00E-27 |
| 92E5 | ++ | 0 | FOXG_08593 | hypothetical protein FG05092.1 | *Gibberella zeae* | 0E |
| 92E5 | ++ | 0 | FOXG_08595 | Leucine carboxyl methyltransferase 1 | *Aspergillus fumigatus* | 4.00E-69 |
| 96H7 | +/- | 0.5 | FOXG_07841 | formate dehydrogenase (NAD-dependent formate dehydrogenase) | *Gibberella zeae* | 0E |
| 100D8 | - | 0 | FOXG_04227 | hypothetical protein FG01907.1 | *Gibberella zeae* | 0E |
| 101E1 | - | 0.1 | FOXG_10510 | Ryp1 | *Ajellomyces capsulatus* | 2.00E-67 |

^a^ -, no growth phenotype; +/-, slightly to severely reduced growth on one or several of the media tested; +, slightly reduced growth on all media tested; ++, severely reduced growth on all media tested; +++, no growth on all media tested, except PDA.
