# Supplementary material for: Insight into the molecular requirements for pathogenicity of Fusarium oxysporum f. sp. lycopersici through large-scale insertional mutagenesis
Source: Genome Biol. 2009 Jan 9;10(1):R4. doi: 10.1186/gb-2009-10-1-r4 (PMC2687792; doi:10.1186/gb-2009-10-1-r4)
Supplement: Additional data file 2 — Pathogenicity mutants with a T-DNA insertion within 500 bp up- or 200 bp downstream of an ORF. [file gb-2009-10-1-r4-S2.doc]

Table S2. Pathogenicity mutants with a T-DNA insertion within 500 bp up- or 200 bp downstream of an ORF.

| mutant ID number | growth phenotype^a^ | pathogenicity phenotype | locus | blast hits |  |  |
| --- | --- | --- | --- | --- | --- | --- |
|  |  | (disease index) |  | description | organism | *E*-value |
| 8E1 | + | 0.4 | FOXG_13733 | mannose-6-phosphate isomerase | *Neurospora crassa* | 1.00E-173 |
| 15G3 | - | 0.1 | unrecognized ORF 232349-233401 - | predicted protein | *Chaetomium globosum* | 6.00E-07 |
| 17A10 | - | 1.1 | FOXG_05187 and | chromatin remodelling complex ATPase chain ISW1 | *Neurospora crassa* | 0E |
|  |  |  | FOXG_05188 | hypothetical protein FG10270.1 | *Gibberella zeae* | 8.00E-115 |
| 19E11 | - | 0 | FOXG_00567 | FAD binding domain protein | *Neosartorya fischeri* | 0E |
| 20G8 | - | 0.6 | FOXG_08864 | WD domain protein | *Neosartorya fischeri* | 0E |
| 23A11 | - | 1.2 | FOXG_00599 | hypothetical protein FG00455.1 | *Gibberella zeae* | 7.00E-113 |
| 25C1 | - | 0 | FOXG_03595 and | RRM domain protein | *Aspergillus fumigatus* | 9.00E-49 |
|  |  |  | FOXG_03596 | protein transport protein SEC61 gamma subunit-like protein | *Magnaporthe grisea* | 9.00E-26 |
| 31E10 | + | 0.6 | FOXG_13757 | manganese superoxide dismutase | *Cordyceps militaris* | 1.00E-94 |
| 42D3 | - | 0.9 | FOXG_00163 | hypothetical protein NCU07505 | *Neurospora crassa* | 7.00E-100 |
| 46A1 | - | 0.4 | FOXG_13429 and | FAD binding domain protein | *Neosartorya fischeri* | 5.00E-77 |
|  |  |  | FOXG_13430 | esterase | *Neosartorya fischeri* | 3.00E-44 |
| 46D3 | - | 1.7 | FOXG_00990 | ABC bile acid transporter | *Aspergillus fumigatus* | 0E |
| 46D7 | - | 2.0 | FOXG_03318 | transcriptional regulator Cti6 | *Neosartorya fischeri* | 2.00E-67 |
| 51D12 | - | 0.3 | FOXG_05914 and | hypothetical protein FG09304.1 | *Gibberella zeae* | 0E |
|  |  |  | FOXG_05915 | plasma membrane zinc ion transporter | *Aspergillus clavatus* | 2.00E-125 |
| 64C7 | - | 1.2 | FOXG_08653 | pyridoxine | *Aspergillus clavatus* | 6.00E-78 |
| 66A3 | + | 0.7 | FOXG_08052 | RINT-1 family protein | *Neosartorya fischeri* | 1.00E-133 |
| 72C7 | +/- | 0 | FOXG_06177 | 3-carboxy-cis,cis-muconate cyclase | *Magnaporthe grisea* | 8.00E-72 |
| 72E5 | - | 0.1 | FOXG_10632 | endosomal sorting complex protein TSG101 | *Aspergillus clavatus* | 5.00E-85 |
| 73B7 | +/- | 0 | FOXG_04162 | class V chitin synthase | *Fusarium oxysporum* | 0E |
| 75G1 | +/- | 1.1 | FOXG_05173 and | proteasome subunit alpha type 6 | *Neurospora crassa* | 1.00E-132 |
|  |  |  | FOXG_05174 | hypothetical protein FG10256.1 | *Gibberella zeae* | 1.00E-128 |
| 80H1 | - | 0.6 | FOXG_04880 | hypothetical protein AN8479.2 | *Aspergillus nidulans* | 0E |
| 86A9 | + | 0.1 | FOXG_09637 | arrestin (or S-antigen), N-terminal domain protein | *Aspergillus fumigatus* | 7.00E-21 |
| 86A9 | + | 0.1 | FOXG_02054 | DUF1183 domain protein | *Aspergillus clavatus* | 2.00E-34 |
| 88B3 | + | 0.6 | FOXG_07874 | L-threo-3-deoxy-hexulosonate aldolase | *Hypocrea jecorina* | 8.00E-131 |
| 100D8 | - | 0 | FOXG_08531 and | mediator of RNA polymerase II transcription subunit 21 | *Saccharomyces cerevisiae* | 9.00E-15 |
|  |  |  | FOXG_08532 | translocation protein Sec62 | *Aspergillus fumigatus* | 2.00E-79 |
| 100G8 | +/- | 1.4 | FOXG_06378 | Zn(II)2Cys6 transcription factor Fow2 | *Fusarium oxysporum* | 0E |

^a^ -, no growth phenotype; +/-, slightly to severely reduced growth on one or several of the media tested; +, slightly reduced growth on all media tested; ++, severely reduced growth on all media tested; +++, no growth on all media tested, except PDA.
