# Supplementary material for: Insight into the molecular requirements for pathogenicity of Fusarium oxysporum f. sp. lycopersici through large-scale insertional mutagenesis
Source: Genome Biol. 2009 Jan 9;10(1):R4. doi: 10.1186/gb-2009-10-1-r4 (PMC2687792; doi:10.1186/gb-2009-10-1-r4)
Supplement: Additional data file 3 — Pathogenicity mutants with a T-DNA insertion within 1,000-500 bp up- or 200-1,000 bp downstream of an ORF. [file gb-2009-10-1-r4-S3.doc]

Table S3. Pathogenicity mutant with a T-DNA insertion within 1,000-500 bp up- or 200-1,000 bp downstream of an ORF.

| mutant ID number | growth phenotype^a^ | pathogenicity phenotype | locus | blast hits |  |  |
| --- | --- | --- | --- | --- | --- | --- |
|  |  | (disease index) |  | description | organism | *E*-value |
| 7H8 | - | 1.2 | FOXG_06378 | Zn(II)2Cys6 transcription factor Fow2 | *Fusarium oxysporum* | 0E |
| 10D1 | +/- | 1.1 | FOXG_08511 | TAM domain methyltransferase | *Neosartorya fischeri* | 4.00E-13 |
| 14E3 | +++ | 0 | FOXG_11153 | bZIP transcription factor | *Aspergillus clavatus* | 2.00E-64 |
| 15G3 | - | 0.1 | FOXG_02717 | hypothetical protein FG07821.1 | *Gibberella zeae* | 1.00E-82 |
| 17A10 | - | 1,05 | FOXG_01226 | Ankyrin and HET domain protein | *Aspergillus fumigatus* | 1.00E-07 |
| 17G9 | +/- | 2.7 | FOXG_11060 | related to lipoyltransferase | *Neurospora crassa* | 3.00E-99 |
| 18A1 | +/- | 0.2 | FOXG_11632 and | amidase family protein | *Aspergillus clavatus* | 0E |
|  |  |  | FOXG_11633 | hypothetical protein FG04021.1 | *Gibberella zeae* | 4.00E-19 |
| 18C4 | - | 0.2 | FOXG_08602 | spherulin | *Neosartorya fischeri* | 2.00E-66 |
| 22F2 | + | 0 | FOXG_01298 | COP9 signalosome subunit 2 (CsnB) | *Aspergillus clavatus* | 0E |
| 31E10 | + | 0.6 | FOXG_00814 | succinate-semialdehyde dehydrogenase NADP+ | *Chaetomium globosum* | 0E |
| 39F6 | - | 0.2 | FOXG_11416 | hypothetical protein FG04213.1 | *Gibberella zeae* | 6.00E-37 |
| 42D3 | - | 0.9 | FOXG_00164 | Autophagy-related protein 22 | *Neurospora crassa* | 0E |
| 44D2 | +/- | 0.6 | FOXG_00090 | phosphate transporter | *Aspergillus clavatus* | 7.00E-83 |
| 48A10 | - | 0 | FOXG_10508 and | phosphatidyl synthase | *Aspergillus clavatus* | 2.00E-33 |
|  |  |  | FOXG_10509 | tyrosyl-DNA phosphodiesterase domain protein | *Aspergillus clavatus* | 2.00E-88 |
| 50C7 | - | 0 | FOXG_01836 | protein-tyrosine phosphatase | *Aspergillus clavatus* | 3.00E-65 |
| 51D10 | - | 1 | FOXG_05014 | hypothetical protein FG10386.1 | *Gibberella zeae* |  |
| 54E10 | - | 1.9 | FOXG_07866 | C6 zinc finger domain protein | *Aspergillus clavatus* | 7.00E-08 |
| 56E4 | - | 0.7 | FOXG_14218 | hypothetical protein FG05307.1 | *Gibberella zeae* | 0E |
| 64C7 | - | 1.2 | FOXG_08652 | Pyridoxine biosynthesis protein PDX1 | *Cercospora nicotianae* | 1.00E-136 |
| 66E12 | + | 0.9 | FOXG_07392 | hypothetical protein FG04661.1 | *Gibberella zeae* | 2.00E-59 |
| 67G4 | - | 0.1 | FOXG_04261 | predicted protein | *Magnaporthe grisea* | 2.00E-72 |
| 67G4 | - | 0.1 | FOXG_03830 | MFS transporter (Mch2) | *Neosartorya fischeri* | 7.00E-27 |
| 69C3 | +/- | 0.1 | FOXG_11097 and | calcium permease | *Botryotinia fuckeliana* | 0E |
|  |  |  | FOXG_11098 | 26S protease regulatory subunit 8 | *Neurospora crassa* | 0E |
| 69E1 | +/- | 0 | FOXG_06082 | no significant hits |  |  |
| 74C9 | +/- | 0.5 | FOXG_07640 | predicted protein FVEG_04565 | *Fusarium verticillioides* | 5.00E-51 |
| 75H3 | - | 0.1 | FOXG_06177 | 3-carboxy-cis,cis-muconate cyclase | *Magnaporthe grisea* | 8.00E-72 |
| 90C8 | +/- | 0.7 | FOXG_08146 | pyruvate decarboxylase | *Fusarium oxysporum* | 0E |

^a^ -, no growth phenotype; +/-, slightly to severely reduced growth on one or several of the media tested; +, slightly reduced growth on all media tested; ++, severely reduced growth on all media tested; +++, no growth on all media tested, except PDA.
