# Supplementary material for: Insight into the molecular requirements for pathogenicity of Fusarium oxysporum f. sp. lycopersici through large-scale insertional mutagenesis
Source: Genome Biol. 2009 Jan 9;10(1):R4. doi: 10.1186/gb-2009-10-1-r4 (PMC2687792; doi:10.1186/gb-2009-10-1-r4)
Supplement: Additional data file 4 — Iintergenic regions are defined as 3,000-1,000 bp up- or downstream of an ORF. [file gb-2009-10-1-r4-S4.doc]

Table S4. Pathogenicity mutants with a T-DNA insertion in ‘intergenic’ regions (defined as 3,000-1,000 bp up- or donwstream of an ORF).

| mutant ID number | growth phenotype^a^ | pathogenicity phenotype | locus | blast hits |  |  |
| --- | --- | --- | --- | --- | --- | --- |
|  |  | (disease index) |  | description | organism | *E*-value |
| 8H8 | - | 1.6 | FOXG_10326 | hypothetical protein FG02719.1 | *Gibberella zeae* | 1.00E-63 |
| 17A10 | - | 1.1 | FOXG_01225 | histidine acid phosphatase | *Neosartorya fischeri* | 4.00E-64 |
| 20F9 | + | 0.1 | FOXG_17539 (contig 53) = FOXG_12401.2 (contig 18) and | no significant hits |  |  |
|  |  |  | unrecgonized ORF (conting 53) = unrecognized ORF (contig 18) | hypothetical protein FG06888.1 | *Gibberella zeae* | 5.00E-110 |
| 20G8 | - | 0.6 | FOXG_08790 | no significant hits |  |  |
| 36B3 | ++ | 0 | FOXG_06304 and | predicted protein FVEG_04155 | *Fusarium verticillioides* | 2.00E-20 |
|  |  |  | FOXG_06305 | vacuolar protein sorting-associated protein vps17 | *Aspergillus fumigatus* | 0E |
| 44A7 | +/- | 0.2 | FOXG_09327 and | hypothetical protein FG05563.1 | *Gibberella zeae* | 0E |
|  |  |  | FOXG_09328 | hypothetical protein FG05552.1 | *Gibberella zeae* | 1.00E-16 |
| 54A4 | - | 0.4 | FOXG_06211 | calcium P-type ATPase NCA-2 | *Neurospora crassa* | 0E |
| 56E4 | - | 0.7 | FOXG_14219 | predicted protein | *Magnaporthe grisea* | 5.00E-30 |
| 63E7 | - | 1.8 | FOXG_13873 | putative histidine phosphotransferase HPT1p | *Gibberella moniliformis* | 6.00E-74 |
| 70G11 | - | 2.0 | FOXG_04163 | chitin synthase 6 ChsVb | *Fusarium oxysporum* | 0E |
| 81H8 | - | 1.1 | FOXG_05278 | transcription factor involved in conidiation StuA | *Fusarium oxysporum* | 0E |

^a^ -, no growth phenotype; +/-, slightly to severely reduced growth on one or several of the media tested; +, slightly reduced growth on all media tested; ++, severely reduced growth on all media tested; +++, no growth on all media tested, except PDA.
