# Supplementary material for: Insight into the molecular requirements for pathogenicity of Fusarium oxysporum f. sp. lycopersici through large-scale insertional mutagenesis
Source: Genome Biol. 2009 Jan 9;10(1):R4. doi: 10.1186/gb-2009-10-1-r4 (PMC2687792; doi:10.1186/gb-2009-10-1-r4)
Supplement: Additional data file 5 — Growth of the pex mutants is disturbed on minimal medium and fatty acids. [file gb-2009-10-1-r4-S5.pdf]

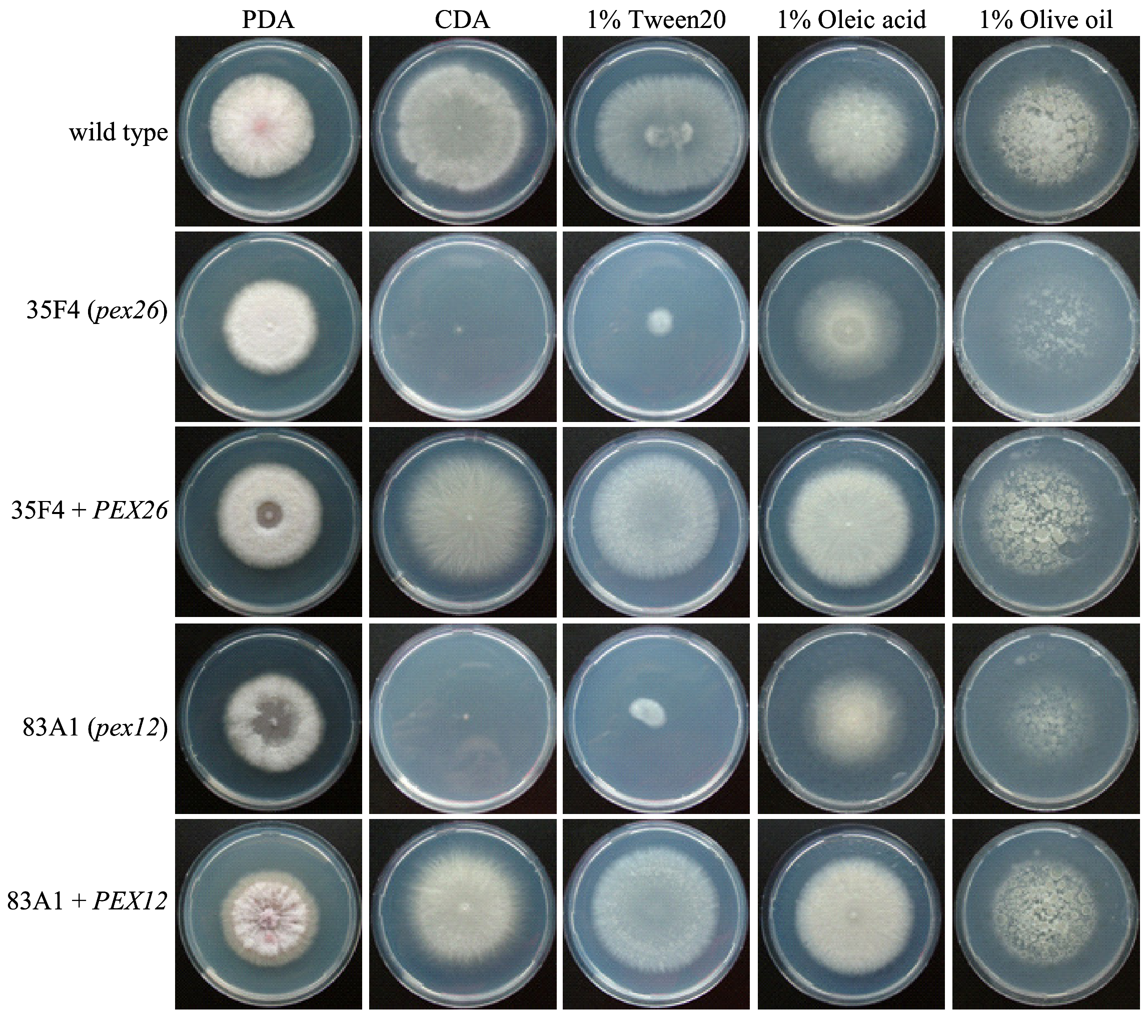

Fig. S1 Growth of the *pex* mutants on rich medium (PDA), minimal medium (CDA) and fatty acids (in medium supplemented with Yeast Nitrogen Base).
