# Supplementary material for: Insight into the molecular requirements for pathogenicity of Fusarium oxysporum f. sp. lycopersici through large-scale insertional mutagenesis
Source: Genome Biol. 2009 Jan 9;10(1):R4. doi: 10.1186/gb-2009-10-1-r4 (PMC2687792; doi:10.1186/gb-2009-10-1-r4)
Supplement: Additional data file 6 — Method and analysis of transformants complemented with FOXG_02084, FOXG_08300 or FOXG_05013. [file gb-2009-10-1-r4-S6.pdf]

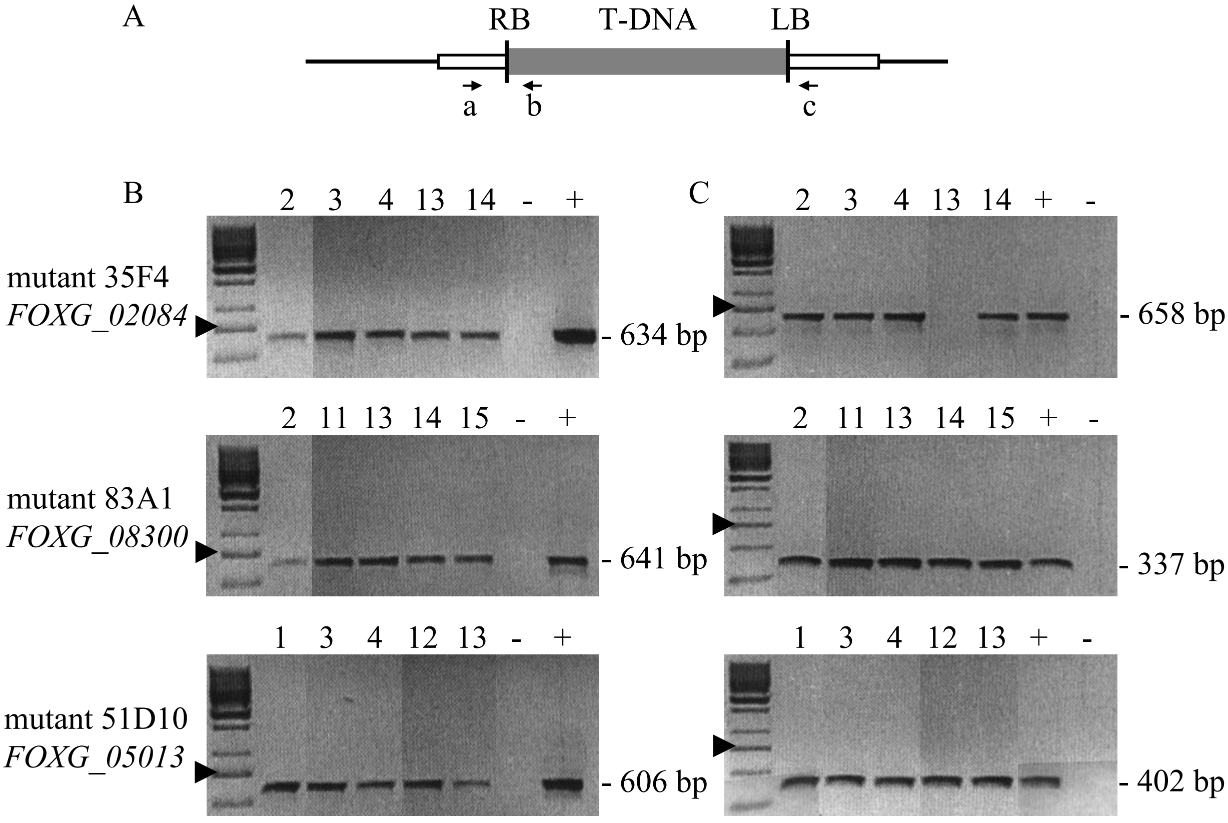


Supplementary figure S2. Analysis of transformants complemented with *FOXG_02084*, *FOXG_08300* or *FOXG_05013*. A, Schematic representation of the T-DNA insertion (not drawn to scale). The open boxes represent the open reading frame disrupted by the T-DNA insertion. Primers a and b were used to check for the presence of the T-DNA. The forward primer is positioned on the chromosomal DNA in front of the right T-DNA border (RB) and the reverse primer is positioned on the T-DNA. Primers a and c were used to check for the presence of the complementation construct and both primers are positioned on the ORF of the disrupted gene. Due to the disruption this primer pair wil not result in a PCR product in the original insertion mutant, since the product size wil be over 6 kb and wil not be amplified under the PCR conditions used. RB, right border. LB, left border. B, Verification of the presence of the complementation construct by PCR. -, water control. +, positive control. Primers FP1694-FP1766, FP1703-FP1767 and FP1677-FP1768 were used in the PCR on transformants complemented with *FOXG_02084*, *FOXG_08300* or *FOXG_05013*, respectively. C, Verification of the presence of the T-DNA by PCR. -, water control. +, positive control. Primers used in the PCR were FP743-FP1694, FP743-FP1703 and FP743-FP1677 on transformants complemented with *FOXG_02084*, *FOXG_08300* or *FOXG_05013*, respectively. In transformant 13 of the complemented 35F4 mutant a rearrangement on the T-DNA had apparently occurred. Since this transformant still contained the complementation construct, it was used in the bioassay. ►, indicates the 750 bp fragment of the 1 kb DNA ladder (Fermentas). The numbers above the panels indicate the transformants used in the bioassays. The figures are composed from different parts of an ethidium bromide gel, which results in minor colour differences.
