# Supplementary material for: Insight into the molecular requirements for pathogenicity of Fusarium oxysporum f. sp. lycopersici through large-scale insertional mutagenesis
Source: Genome Biol. 2009 Jan 9;10(1):R4. doi: 10.1186/gb-2009-10-1-r4 (PMC2687792; doi:10.1186/gb-2009-10-1-r4)
Supplement: Additional data file 8 — Method and analysis of transformants deleted for FOXG_03318. [file gb-2009-10-1-r4-S8.pdf]

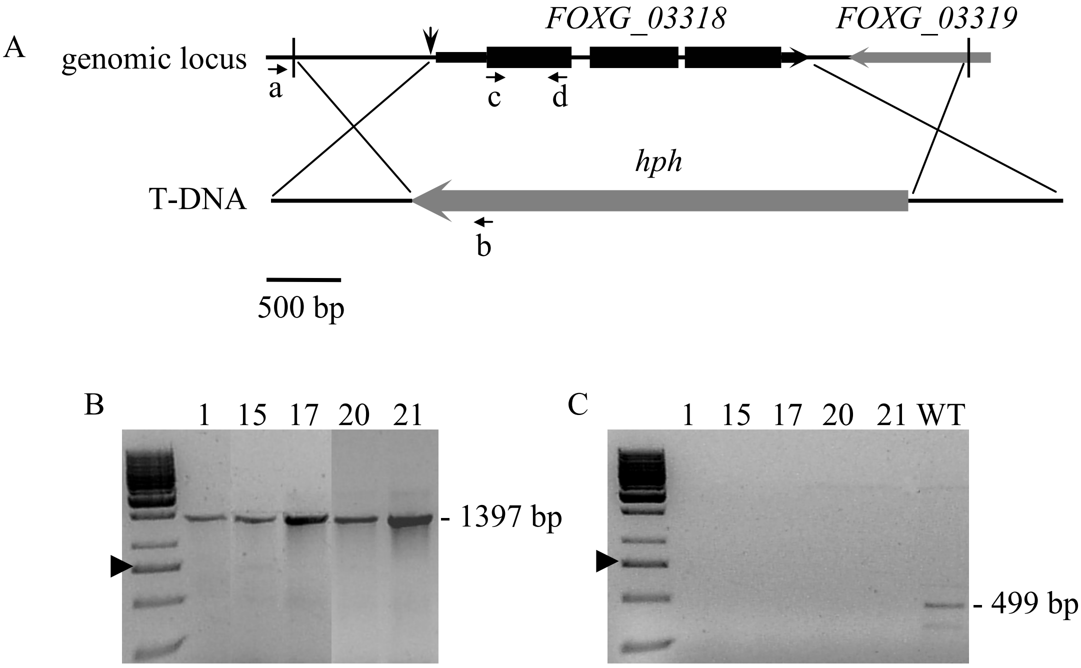

Supplementary figure S4. Analysis of transformants deleted for *FOXG_03318*. A, Schematic representation of the knock-out strategy for F*OXG_03318* drawn to scale. The arrow head indicates the position of the original T-DNA insertion. The small arrows represents the primers used for checking homologous recombination (a and b) and for checking the absence of the open reading frame (c and d). B, Verification of homologous recombination by PCR using primers a (FP1926) and b (FP582). C, Verification of the absence of the open reading frame by PCR using primers c (FP1924) and d (FP1925). The numbers above the panels indicate the transformants used in the bioassay. WT, wild type. ►, indicates the 750 bp fragment of the 1 kb DNA ladder (Fermentas). The figures are composed from different parts of an ethidium bromide gel, which results in minor colour differences.
