# Supplementary material for: Insight into the molecular requirements for pathogenicity of Fusarium oxysporum f. sp. lycopersici through large-scale insertional mutagenesis
Source: Genome Biol. 2009 Jan 9;10(1):R4. doi: 10.1186/gb-2009-10-1-r4 (PMC2687792; doi:10.1186/gb-2009-10-1-r4)
Supplement: Additional data file 11 — Primer sequences used for PCR and sequencing. [file gb-2009-10-1-r4-S11.doc]

Table S5. Primers used in this study.

| primer | Sequence (5’-3’) |
| --- | --- |
| HPH-Fwd2-ApaI | aaa**gggccc**tagggatccacttaacgttac |
| HPH-EndApaI-f | aaa**gggccc**tagagtagatgccggaccgc |
| GFP-f-ApaI | aaa**gggccc**atggtgagcaagggcgaggag |
| GFP-r-ApaI | aaa**gggccc**ttacttgtacagctcgtcc |
| FOXG_05013f-XbaI | gc**tctaga**gttttgtgtttgaagcattaacg |
| FOXG_05013r-AscI | tt**ggcgcgcc**tcatcattctttcaaggcttctc |
| FOXG_05013-f1 | cttcttcgcgatccaccgtc |
| FOXG_05013-f2 | cgccttatagcattgcatcc |
| FOXG_05013-f3 (FP1677) | gagatatttaccttgatcgac |
| FOXG_05013-f4 | ggaggagacacaaagcgtg |
| FOXG_05013-r1 (FP1768) | cacgctttgtgtctcctcc |
| FOXG_02084f-AscI | tt**ggcgcgcc**agaacaaatgcgttgtatgc |
| FOXG_02084r-XbaI | gc**tctaga**tatcctgccgagcctacc |
| FOXG_02084-f1 | agctgtgcccttgaccttag |
| FOXG_02084-f2 (FP1694) | cattctcaagtctttcctg |
| FOXG_02084-f3 | cagaccttaatgcgagtcg |
| FOXG_02084-f4 | gactcaggaacgcaggc |
| FOXG_02084-r1 (FP1766) | cgactcgcattaaggtctg |
| FOXG_08300f-XbaI | gc**tctaga**tatcaccatcaatattgacc |
| FOXG_08300r-AscI | tt**ggcgcgcc**agcagttcgtcttttaattc |
| FOXG_08300-f1 | gtgtcggacatgttgtttg |
| FOXG_08300-f2 | caatggagttcgtcacagc |
| FOXG_08300-f3 (FP1703) | catgcttgctttcaatgtgg |
| FOXG_08300-f4 | gagggtaagtgggagagtg |
| FOXG_08300-r1 (FP1767) | cactctcccacttaccctc |
| FOXG_08602-f3 | ccc**aagctt**gccatgactcgtctcagctc |
| FOXG_08602-r3 | tt**ggcgcgcc**tccaatctcagccggatac |
| FOXG_08602-f4 | gg**ggtacc**gagcatgtgagcgatgtttc |
| FOXG_08602-r4 | cc**ttaattaa**cacggccacagtacacagtc |
| FOXG_03318-f1 | ccc**aagctt**gattcctgtagtaggtatac |
| FOXG_03318-f2 | gg**ggtacc**cagcattttgtttcagtaggtc |
| FOXG_03318-r1 | gc**tctaga**tctcactttgcttgggatac |
| FOXG_03318-r2 | cc**ttaattaa**caacgctctacggttccaag |
| FOXG_09487-f1 | ccc**aagctt**caactcaagctatataaggac |
| FOXG_09487-r1 | gc**tctaga**gtgtggtaaggtagagttgag |
| FOXG_09487-f2 | gg**ggtacc**tatcacagtatctatcgtgac |
| FOXG_09487-r2 | cc**ttaattaa**ccttgtcaagacccgtttac |
| 86A9pro_fw | tt**ggcgcgcc**tggagctgatggtatgtg |
| 86A9pro_rev | gc**tctaga**gctatagggtcgactatacg |
| 86A9term_fw | gg**ggtacc**acggatggtcctccggag |
| 86A9term_rev | cc**ttaattaa**ccgagagcgcaatggaaac |
| FP1920 (FOXG_08602-f6) | cactatgattcaactggaatc |
| FP1978 (TrpC-f5) | cagaatgcacaggtacacttg |
| FP1918 (FOXG_08602-f5) | gctcctcgcaatactcgac |
| FP1919 (FOXG_08602-r5) | gttgaactgtgtgtggatag |
| FP1926 (FOXG_03318-f4) | cacgactgagtgtttggcac |
| FP582 (TrpC-f3) | gactgaggaatccgctcttg |
| FP1924 (FOXG_03318-f3) | caagcacgtctggtcgag |
| FP1925 (FOXG_03318-r3) | catgctgggaaatcgacttac |
| FP1929 (FOXG_09487-f4) | gtgttgcttatcatgagactg |
| FP1927 (FOXG_09487-f3) | gtgagttatcatcattctctc |
| FP1928 (FOXG_09487-r3) | gcatcatcatctggtgtgac |
| FP1914 (FOXG_02054opp-fw) | ggacttgtttcatggttatgg |
| FP1915 (FOXG_02054orf-fw) | ccatcaagctttccgatgtg |
| FP1916 (FOXG_02054orf-rev) | agcctcttgtgctctgtaac |
| LB-f | gttgccgttcttccgaatag |
| RB-r | ggcatgcacatacaaatgga |
| pPK2-LB1 | gtgcctaatgagtgagctaactccc |
| pPK2-LB2 | ctcacattaattgcgttgcgctc |
| pPK2-LB3 | gagcaattcggcgttaattcagt |
| pPK2-RB1^a^ | tggcactggccgtcgttttacaac |
| pPK2-RB2^a^ (FP743) | aacgtcgtgactgggaaaaccct |
| pPK2-RB3^a^ | cccttcccaacagttgcgca |
| AD1^a^ | wagtgnagwancanaga |
| AD2 | tcmagga5gcygcyac |
| AD3 | gtrgcrgc5tcctkga |
| AD4 | ntcgastwtsgwgtt |
| AD6 | ccyacyga5gaggtcm |
| AD7 | ctkgaag5atyatygt |
| AD8 | agktcctycg5cg5tg |
| M13forward | ttcccagtcacgacgttgt |
| M13reverse | cagctatgaccatgattacg |

Restriction sites are indicated in bold letter type.

^a^, Primers developed by Mullins *et al*. [100].
