# Supplementary material for: Insight into the molecular requirements for pathogenicity of Fusarium oxysporum f. sp. lycopersici through large-scale insertional mutagenesis
Source: Genome Biol. 2009 Jan 9;10(1):R4. doi: 10.1186/gb-2009-10-1-r4 (PMC2687792; doi:10.1186/gb-2009-10-1-r4)
Supplement: Additional data file 12 — Conditions used for TAIL-PCR. [file gb-2009-10-1-r4-S12.doc]

Table S6. Conditions used for thermal asymmetric interlaced PCR^a^.

| Primary PCR reaction | Secondary PCR reaction | Tertiary PCR reaction |
| --- | --- | --- |
| 95°C: 2 min | 94°C: 30 sec | 94°C: 30 sec |
| 94°C: 1 min | 66°C: 1 min | 94°C: 15 sec |
| 62°C: 1 min | 72°C: 2 min | 42°C: 15 sec |
| 72°C: 2 min 30 sec | 94°C: 10 sec | 72°C: 2 min |
| step 2-4: 4 times | 66°C: 1 min | step 2-4: 29 times |
| 94°C: 1 min | 72°C: 2 min | 72°C: 5 min |
| 25°C: 3 min | 94°C: 10 sec |  |
| ramp to 75°C over 3 min | 44°C: 1 min |  |
| 72°C: 3 min | 72°C: 2 min |  |
| 94°C: 30 sec | step 1-9: 14 times |  |
| 68°C: 1 min | 72°C: 5 min |  |
| 72°C: 2 min, 30 sec |  |  |
| 94°C: 30 sec |  |  |
| 68°C: 1 min |  |  |
| 72°C: 2 min 30 sec |  |  |
| 94°C: 30 sec |  |  |
| 44°C: 1 min |  |  |
| 72°C: 2 min 30 sec |  |  |
| step 10-18: 14 times |  |  |
| 72°C: 5 min |  |  |
| 4°C: 1 min |  |  |

^a^, PCR conditions adapted from Mullins *et al*. [98].
